# PertInInt: An Integrative, Analytical Approach to Rapidly Uncover Cancer Driver Genes with Perturbed Interactions and Functionalities

**DOI:** 10.1016/j.cels.2020.06.005

**Published:** 2020-07-14

**Authors:** Shilpa Nadimpalli Kobren, Bernard Chazelle, Mona Singh

**Affiliations:** 1 Department of Biomedical Informatics, Harvard Medical School, Boston, MA, USA; 2 Department of Computer Science, Princeton University, Princeton, NJ, USA; 3 Lewis-Sigler Institute for Integrative Genomics, Princeton University, Princeton, NJ, USA; 4 Lead Contact

## Abstract

A major challenge in cancer genomics is to identify genes with functional roles in cancer and uncover their mechanisms of action. We introduce an integrative framework that identifies cancer-relevant genes by pinpointing those whose interaction or other functional sites are enriched in somatic mutations across tumors. We derive analytical calculations that enable us to avoid time-prohibitive permutation-based significance tests, making it computationally feasible to simultaneously consider multiple measures of protein site functionality. Our accompanying software, PertInInt, combines knowledge about sites participating in interactions with DNA, RNA, peptides, ions, or small molecules with domain, evolutionary conservation, and gene-level mutation data. When applied to 10,037 tumor samples, PertInInt uncovers both known and newly predicted cancer genes, while additionally revealing what types of interactions or other functionalities are disrupted. PertInInt’s analysis demonstrates that somatic mutations are frequently enriched in interaction sites and domains and implicates interaction perturbation as a pervasive cancer-driving event.

## In Brief

A fast, analytical framework called PertInInt enables efficient integration of multiple measures of protein site functionality—including interaction, domain, and evolutionary conservation—with gene-level mutation data in order to rapidly detect cancer driver genes along with their disrupted functionalities.

## Graphical Abstract

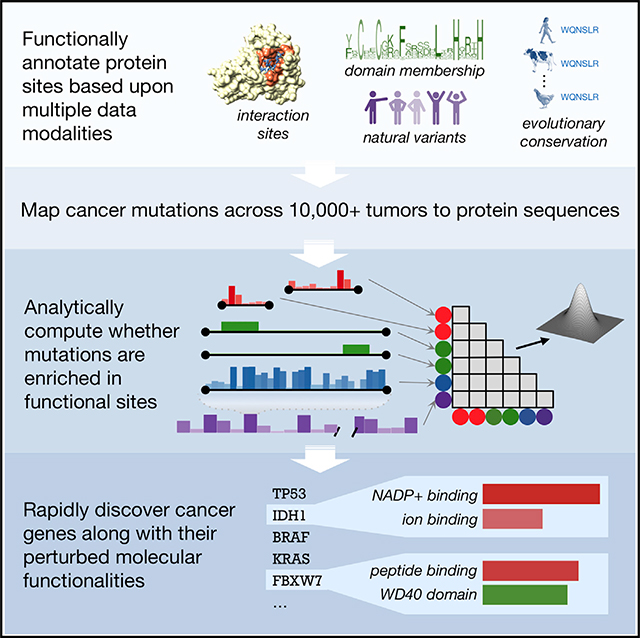

## INTRODUCTION

Large-scale, concerted oncogenomic consortia, such as the Cancer Genome Atlas (TCGA) and International Cancer Genome Consortium (ICGC), have sequenced an unprecedented number of tumor genomes from thousands of patients across tens of cancer types (International Cancer Genome Consortium et al., 2010; TCGA Research Network et al., 2013). Computational analyses of these datasets promise a revolution in precision oncology with additional insights into the genetic underpinnings of a staggeringly complex and heterogeneous disease (Chin and Gray, 2008). The recent, successful completion of these efforts, heralded as the “end of the beginning” of cancer genomics, has revealed a critical need for new methods that are able both to detect less frequent cancer-driving mutational events as well as to suggest the mechanistic, molecular impact of these mutations (Bailey et al., 2018; The ICGC/TCGA Pan-Cancer Analysis of Whole Genomes Consortium et al., 2020). More broadly, the comprehensive detection of cancer-driving mutational events, coupled with an understanding of their biological mechanism of action, has the potential to expand our knowledge of altered cellular processes in tumors, to reveal actionable, genetic similarities between different cancer types, to inform how evolving, heterogeneous populations of tumor cells may impact therapeutic efficacy, and to further translational research and inform downstream clinical treatments (McGranahan and Swanton, 2017; Vogelstein et al., 2013).

Despite considerable efforts, the crucial first step toward these goals—differentiating the small fraction of somatic mutations with functional roles in cancer (“drivers”) from the preponderance of neutral “passenger” mutations—still poses a substantial computational obstacle (Porta-Pardo et al., 2017). While initial attempts to uncover cancer drivers at the gene level based on frequency of mutation across tumor samples have been fruitful (Dees et al., 2012; Lawrence et al., 2013), such gene-centric, recurrence-based approaches are inherently unable to detect infrequently mutated driver genes and also cannot distinguish among mutations within the same gene that may lead to distinct tumor phenotypes or clinical responses (Torkamani and Schork, 2008). Indeed, different positions within genes can contribute in varying degrees to different molecular functionalities; mutations falling within different gene positions can therefore have unequal impacts. In order to address the critical need to detect and interpret rare mutational driver events, an emerging class of “subgene” level approaches consider somatic mutations affecting genes within the context of information known about specific sites within their encoded proteins (Porta-Pardo et al., 2017). Existing subgene-level methods have derived such protein site functionality information from analyses of evolutionary conservation (Adzhubei et al., 2010; Ng and Henikoff, 2003; Reva et al., 2011), three-dimensional structure (Gao et al., 2017; Kamburov et al., 2015; Niu et al., 2016; Porta-Pardo et al., 2015; Ryslik et al., 2014a; Tokheim et al., 2016a), domains (Munro et al., 2018; Peterson et al., 2017; Porta-Pardo and Godzik, 2014), or post-translational modification (Reimand and Bader, 2013; Zhao et al., 2017). These methods, however, tend to identify cancer genes by considering whether somatic mutations alter just a single type of functionality (e.g., determining whether mutations are enriched only within protein domains [Porta-Pardo and Godzik, 2014] or only within phosphorylation sites [Reimand and Bader, 2013]), whereas somatic mutations within putative driver genes have been found to disrupt a broad range of protein functionalities. On the other hand, machine learning approaches to classify cancer drivers incorporate multiple types of information, but due to their “black box” nature, mechanistic interpretations of their predictions are not possible (Carter et al., 2009; Shihab et al., 2013).

We and others have previously demonstrated that detecting proteins that harbor somatic mutations in their interaction interfaces is a particularly effective approach to pinpoint infrequent driver mutations as well as reason about their molecular impacts and therapeutic sensitivities (Engin et al., 2016; Ghersi and Singh, 2014; Gress et al., 2016; Kamburov et al., 2015; Kar et al., 2009; Nishi et al., 2013; Porta-Pardo et al., 2015; Stehr et al., 2011). Indeed, several cancer driver genes, including *TP53* and *IDH1*, are well known to harbor mutations within their interaction sites (Nishi et al., 2013). While traditionally interaction sites have been identified directly for the small fraction of human genes with actual or modeled co-complex structures, we have recently developed a domain-based approach that accurately detects residues that interact with DNA, RNA, peptides, ions, or small molecules across 63% of human genes (Kobren and Singh, 2019). A robust computational framework that utilizes this vastly expanded knowledge-base about interaction sites and explicitly integrates it with additional lines of evidence regarding subgene functionality would provide a powerful new approach not only to detect but also to interpret a wide range of mutations driving protein dysfunction in cancer.

Here, we introduce a fast, interpretable, and easily extendable framework that enables us to uncover whether somatic mutations within genes are enriched in sites associated with high measures of “functionality” as determined by multiple, possibly correlated, lines of evidence. Our implementation PertInInt (pronounced “pertinent,” perturbed in interactions) incorporates interaction site information, along with evolutionary conservation and domain membership information, as each of these measures informs which sites are important for protein functioning. We derive analytical calculations that obviate the need to perform time-prohibitive permutation-based significance tests, thereby making it feasible to integrate, in a principled manner, these distinct measures of subgene-level functionality. Further, we extend our framework to consider whole-gene mutation rates, as genes that are recurrently mutated across tumors are often found to be causally implicated in cancers (Forbes et al., 2010). While other approaches have combined the output of multiple programs post hoc (e.g., Bailey et al., 2018), PertInInt integrates multiple alternate sources of subgene resolution data with whole-gene mutational frequency within a single unifying framework in order to detect, evaluate, and infer the molecular impact of patterns of somatic mutations within all human genes.

We apply PertInInt to somatic missense mutation data arising from 10,037 tumor samples across 33 cancer types to identify genes with the most enriched mutational patterns. We find that while each source of information—interaction, domain, evolutionary conservation, and whole-gene mutation frequency—is individually predictive of cancer genes, PertInInt uncovers more comprehensive sets of cancer-relevant genes when considering all sources of information together. We demonstrate that PertInInt is able to identify even those cancer genes with relatively low overall mutation rates, and that PertInInt readily outperforms previous methods while revealing whether and what type of interaction potential is perturbed. PertInInt finds that numerous known oncogenes and tumor suppressors have an enrichment of somatic mutations within their interaction interfaces and, in addition, predicts new cancer-relevant genes along with their altered interaction functionalities. Altogether, PertInInt provides a highly effective integrative framework to analyze large-scale cancer somatic mutation data and further our understanding of the molecular mechanisms driving cancers.

## RESULTS

### Overview of the PertInInt Framework

**Figure 1.**
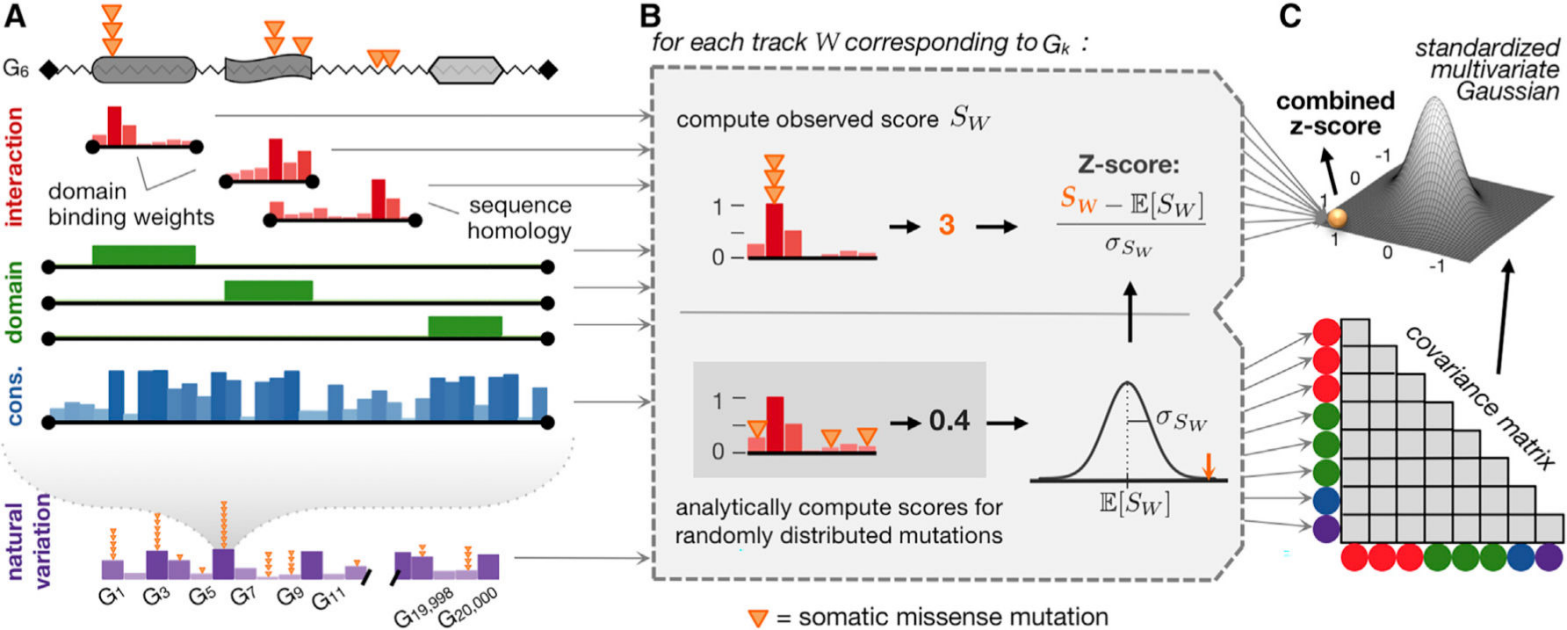
PertInInt Uncovers Cancer Driver Genes by Integrating Per-Site Interaction, Domain, and Conservation Information with Whole-Gene Mutation Frequency Data (A) Somatic mutations (orange triangles) found across sequenced tumors that affect a protein sequence (jagged line) with three domains (gray regions) are evaluated with respect to different measures of functionality, each represented as a “track.” In interaction tracks (red), positions that are more likely to participate in ligand interactions have higher weights (vertical bars). Interaction tracks arise from domain-based binding potential calculations (Kobren and Singh, 2019) (top two red tracks, each covering the length of the respective domain) or homology modeling (Ghersi and Singh, 2014) (bottom red track, covering the length of the modeled region). Domain tracks (green) specify which residues within a protein are part of a specific domain by 0/1 positional weights; here we have a track for each domain within the sequence. The conservation track (blue) weights each position by its evolutionary conservation across species. The natural variation track (purple) models how much each gene varies across healthy populations; here the height of the vertical bars indicates the background mutation probability rather than a per-gene weight, which is 1 for the gene being considered and 0 otherwise. Figure S1 gives further intuition about how these track weights are determined. (B) For each track *W*, we compute the score *S*_*W*_ of the observed somatic mutations as the sum of the track weights for the positions where they appear (top). To determine whether this score is higher than expected, we consider a model where somatic mutations are shuffled across the positions of the track, and the expected score $\left(E\left[S_{W}\right]\right)$ and the standard deviation of the scores $\left(\sigma_{S_{W}}\right)$ are computed and used to estimate per-track *Z* scores (bottom); note that in our framework these values are computed analytically instead of relying on the shuffles. (C) *Z* scores for all tracks are combined after analytically determining a background covariance model.

PertInInt aggregates somatic mutational data observed across tumor samples and identifies for each gene whether certain types of its functional sites are enriched in somatic mutations and/or whether the gene exhibits a high mutation rate across its length. We briefly overview our approach next (see also Figure 1); more details can be found in the STAR Methods section and Figure S1.

Different measures of protein site functionality are modeled using distinct “tracks” where each position within a track has a corresponding 0 to 1 weight that reflects its importance with respect to the functionality being considered (Figure 1A). Though any type of annotated functional region can be incorporated into our framework, here, we consider four specific types of tracks. First, “interaction tracks” model various protein–ligand interaction interfaces, where higher positional weights indicate that those positions are more likely to participate in interactions with a ligand; each interaction track corresponds to the subset of protein positions where we have any knowledge about ligand-binding potential (Figures S1A–S1C). Second, “domain tracks” span the length of the protein and simply identify portions of the protein sequence that correspond to the domain of interest; weights are 1 for amino acid positions within the domain and 0 elsewhere (Figure S1D). Third, the “conservation track” is also the length of the protein sequence, and the weight of each position measures its conservation across vertebrate homologs; higher weights correspond to positions under more evolutionary constraint (Figure S1E). Finally, to determine whether a gene as a whole has more mutations than expected, we extend our framework to incorporate the “natural variation track,” which has a single entry per gene that reflects its background mutation rate, as estimated from the number of variants this gene has across healthy populations (Lek et al., 2016; Przytycki and Singh, 2017) (Figure S1F). Approximately 63% and 90% of human genes have per-site information about interactions or domains respectively, while all genes have per-site conservation values and background gene-level mutation rates. A gene may have numerous interaction and domain tracks (e.g., for different modeled interaction regions and for each of its identified domains) but has only a single conservation and natural variation track.

For each track, we consider the somatic mutations observed across tumor samples that fall within positions of that track and compute a per-track score as the sum of the per-track weights of the positions that each of the mutations fall into (Figure 1B); intuitively, a high score corresponds to the case where a large number of mutations fall within important track positions. To determine whether the score for a track is more than expected by chance, we could shuffle the mutations across the positions of the track, and use the mean and standard deviation computed from these permutations to compute a *Z* score; however, the mean and standard deviation for each track can be computed analytically. For each protein, we next combine the information from each of its tracks. Because tracks can overlap along the length of the protein sequence, and the somatic mutations that fall in each of them can also overlap, these tracks cannot be treated independently. Instead, for the background model we derive an approach to compute the covariance between tracks analytically and then use this covariance matrix to estimate a combined score (Figure 1C; see STAR Methods for derivations). We find that even when considering just a single track, our analytical formulation leads to >7× speedup over *each* empirical permutation (Figure S2). In practice, numerous shuffles are necessary to compute the mean and variance for a single track, and empirical calculations to estimate the covariance across all tracks is prohibitively slow, highlighting the power and necessity of our analytical formulation.

The final per-gene score output by PertInInt considers whether somatic mutations across samples are enriched (1) in positions with high ligand-binding potential for an interaction track, (2) within domain positions for a domain track, (3) within conserved sites for the conservation track, and (4) within the gene overall.

### PertInInt Effectively Identifies Cancer Driver Genes via Integrating Multiple Sources of Information

We run PertInInt on somatic point mutation data aggregated across 10,037 pan-cancer tumor samples and 33 tumor types from TCGA (TCGA Research Network et al., 2013) (Figure S3 and Table S1). PertInInt’s analytical formulation enables the simultaneous consideration of multiple types of biological data regarding protein functionality. However, to first uncover to what extent each source of information—per-site interaction, domain, and conservation information as well as overall gene mutational frequency—is independently useful for identifying cancer-relevant genes, we run PertInInt on the pan-cancer dataset when restricted to each of these track types in turn. To validate the method in the absence of a complete gold standard, as we consider an increasing number of output genes, we compute how “enriched” this set is in genes from the Cancer Gene Census (CGC), a curated list of genes implicated in cancer (Futreal et al., 2004). In particular, enrichment is computed as the ratio between the fraction of CGC genes in the set of top-scoring genes considered (i.e., the precision) and the fraction of CGC genes in the whole set of genes (i.e., the precision you would expect to achieve if genes are randomly ordered).

**Figure 2.**
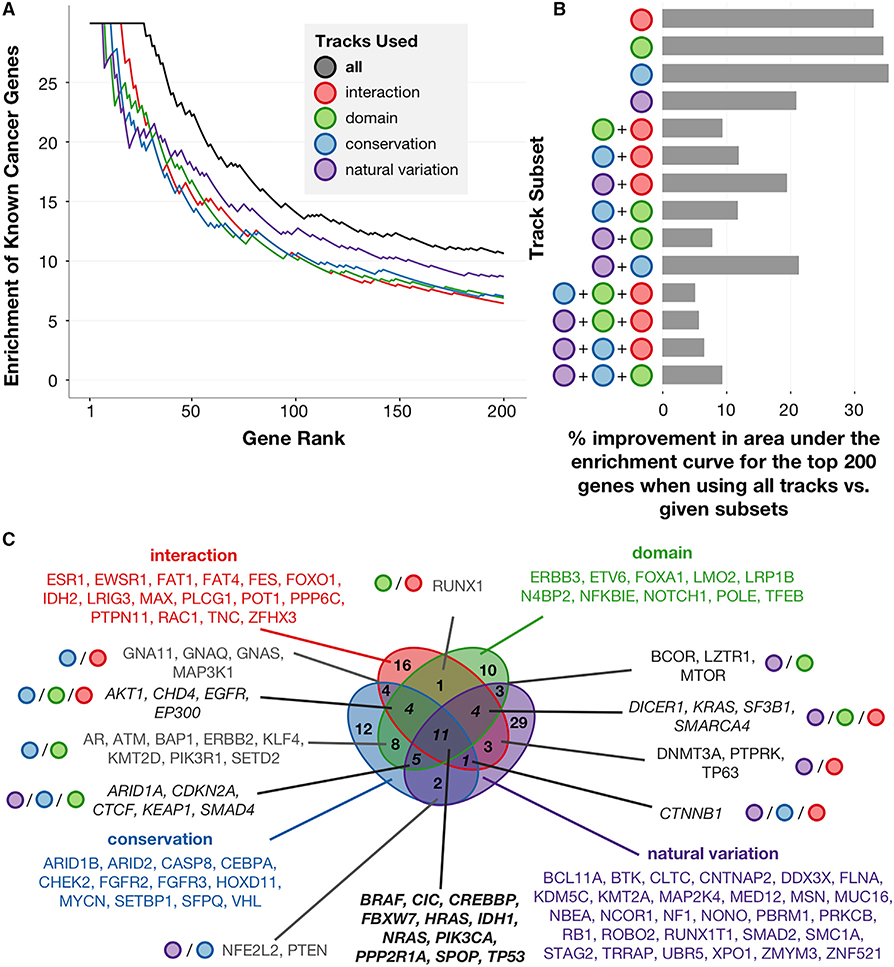
PertInInt Is Highly Effective in Uncovering Cancer Driver Genes Due to Combining Multiple Sources of Information (A) Enrichment of CGC genes (y axis) within a given number of top-scoring genes (x axis) when run on the pan-cancer dataset using all tracks together (black), only interaction tracks (red), only domain tracks (green), only the conservation track (blue), and only the natural variation track (purple). Enrichment is computed as the ratio between the fraction of CGC genes in the set of top scoring genes considered (i.e., the precision) and the fraction of CGC genes in the whole set of genes (~0.0334). While uncovering genes enriched for somatic mutations within only interaction sites, only domain positions, only conserved sites, or only over their lengths each yields cancer-relevant genes, performance is highest when PertInInt uses all sources of information together. (B) Percent improvement in the area under the enrichment curve for the top 200 genes when using all track types versus specific subsets of tracks. PertInInt is more effective in uncovering CGC genes when using all sources of information together than when using any other of the possible subsets of information. (C) Venn diagram showing the overlap of CGC genes detected in the top 200 genes ranked when considering only interaction, only domain, only conservation, or only natural variation tracks. The different sources of information yield distinct yet overlapping sets of cancer genes.

We find that utilizing subsets of only interaction, only domain, only conservation, or only natural variation tracks in turn can recapitulate known CGC genes to varying degrees, with interaction tracks identifying the largest number of known driver genes while maintaining perfect precision relative to other track subsets (Figure 2A). Our integrative framework that incorporates all track types outperforms every version of our algorithm that considers only subsets of information; indeed, considering any two sources of biological information outperforms versions of PertInInt that utilize only one source, and considering any three sources of data tends to improve performance even further (Figure 2B). Attempts to combine per-track scores without accounting for between-track covariance (e.g., by summing or averaging track *Z* scores) not only are incorrect but also perform considerably worse in detecting cancer-relevant genes (Figure S4). Altogether, these results demonstrate the ability of our approach to effectively leverage the distinct contributions of multiple, complementary data sources regarding protein position and whole-gene functionality in order to uncover cancer driver genes. Furthermore, the enrichment of cancer genes among PertInInt’s top predictions remains when considering different gold standards (Figure S5).

We find low overlap between the sets of CGC genes identified when utilizing distinct track types, indicating that mutations within cancer genes tend to target a diverse array of functional elements (Figure 2C). Only a small minority of CGC genes (less than 10%) are identified by all four track types within the top 200 ranked genes. Mutations falling into known tumor suppressor *PTEN*, for instance, tend to hit evolutionarily conserved protein positions but do not alter known inferred interaction interfaces or domain regions more than expected by chance. In contrast, a small molecule-binding pocket in the *IDH2* oncogene is recurrently mutated across cancers, and, thus, it is readily detected using interaction tracks alone but is less significantly ranked when PertInInt is restricted to other functionality data.

### Lowly Mutated Genes Harbor Mutations that Preferentially Alter Functional Sites

**Figure 3.**
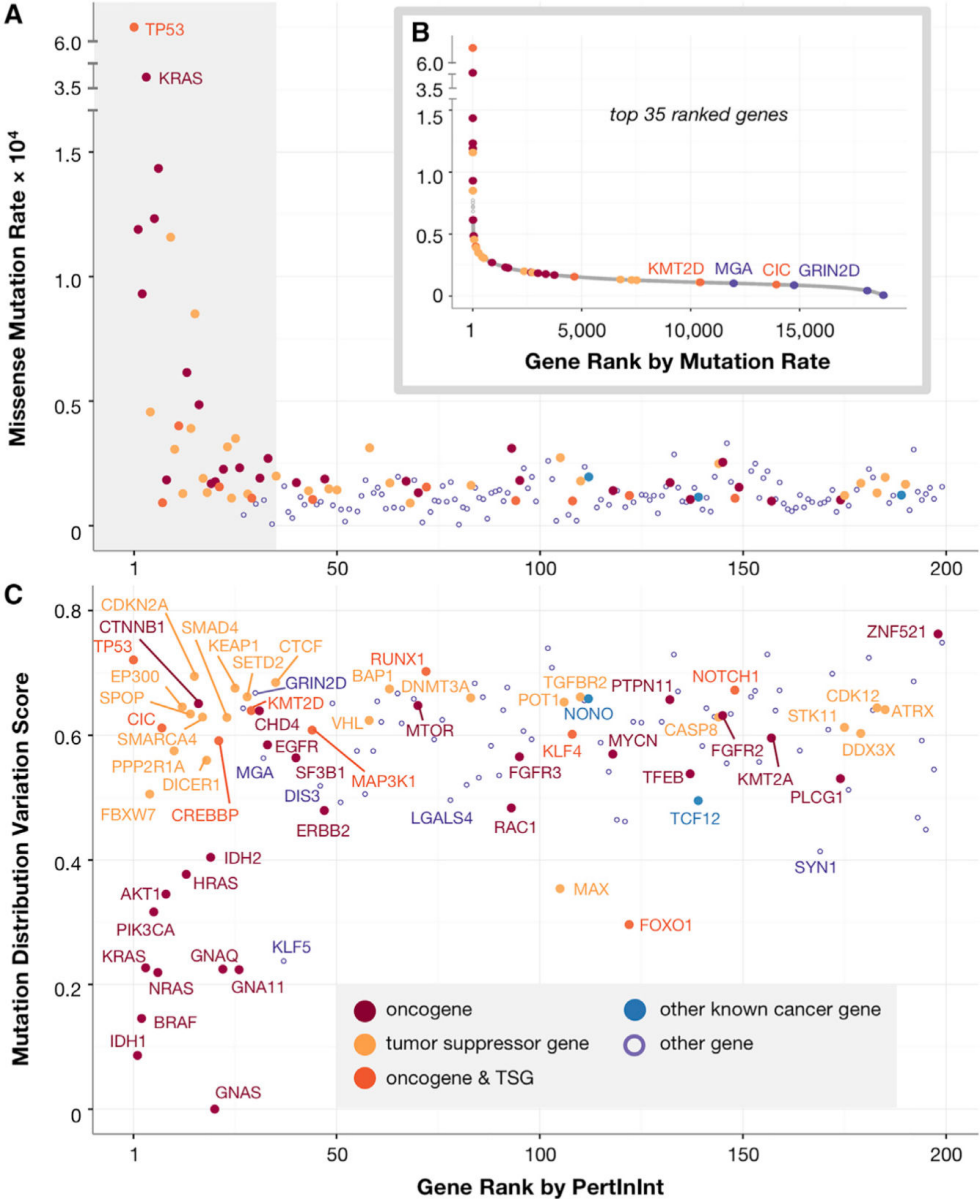
Perturbed Interaction Interfaces across Oncogenes and Tumor Suppressors (A) Shown are the missense mutation rates (y axis) of the top 200 genes ranked by PertInInt (x axis). Top-ranked genes are both highly and infrequently mutated. Genes are colored as in (C). The shaded gray box highlights the plot to 35 genes, which are featured in the part (B) inset. (B) Genes are ordered by their per-tumor-sample missense mutation rate in the pan-cancer dataset (x axis), and their missense mutation rate is given (y axis). PertInInt’s top 35-ranked genes are plotted in color and exhibit a wide range of ranks with respect to mutation rate. Of these, only genes with below-median overall mutation rates and a *Z* score ≥ 1 in at least one interaction track are labeled. (C) For each of the top 200 genes ranked by PertInInt (x axis), for those with a *Z* score ≥ 1 in at least one interaction track, we also analyze the distribution of somatic mutations across interaction sites and compute their normalized Shannon entropy (y axis). These genes contain recurrent (low variation) as well as more distributed (high variation) mutations across their binding interfaces.

We next show that PertInInt’s integrative approach can highlight genes with preferentially altered functional sites that may be lowly mutated overall; such “long tail” driver genes are easily missed by traditional frequency-based driver gene detection approaches. When run on the pan-cancer dataset utilizing all track types, PertInInt ranks highly several such infrequently mutated genes (Figure 3A). Of the top 35 genes ranked by PertInInt on the pan-cancer dataset, we find that 20 have a missense mutation rate less than one-twentieth of the maximum observed mutation rate (Figure 3B). These high-scoring long tail genes include novel genes with potential roles in cancer as well as known driver genes that cannot have been identified based solely on their relative mutation frequency (e.g., *KMT2D* and *CIC*, Figure 3B). Of the 20 highly ranked infrequently mutated genes, 18 harbor perturbed interaction sites, enabling immediate molecular insights regarding their roles in cancer. For example, among long tail genes that are highly ranked by PertInInt but have not yet been identified as cancer relevant, several have an enrichment of mutations in their DNA or small molecule interaction sites (e.g., *MGA* and *GRIN2D*, Figure 3B), in line with previous observations that many cancer driver genes exhibit these types of protein interaction perturbations (Delgado and León, 2006; Jeggo et al., 2016; Raimondi et al., 2017).

### Mutations Are Distributed across Interaction Interfaces

For each protein with a significantly perturbed interaction interface, we next sought to determine whether mutations are found within a small number of interaction sites or across several interaction sites. We consider all sites within the protein with nonzero interaction track weights and use the frequency with which somatic mutations occur within each of them to compute a normalized Shannon entropy (Shannon, 1948). Higher entropies correspond to proteins with mutations spread across many interaction sites, whereas low entropies correspond to mutational patterns that can be uncovered by methods that look for mutation “hotspots” (Chang et al., 2016). As expected, PertInInt highly ranks several oncogenes that have previously been detected by hotspot detection algorithms due to their recurrent mutations in critical interaction positions (e.g., *IDH1*, *BRAF*, and *NRAS*). However, there are also many genes with significantly perturbed interaction interfaces where mutations are spread more widely across their interaction sites (Figure 3C). Known cancer genes *DICER1*, *SMARCA4*, *CREBBP*, and *KMT2D*, for instance, are among the top 35 genes ranked by PertInInt and contain significantly mutated interaction sites (combined score across interaction tracks > 6), each with several interaction sites that together harbor an enriched number of somatic mutations.

Notably, this analysis reveals that the top-ranked genes with significantly perturbed interaction interfaces include both oncogenes and tumor suppressor genes (TSGs), reflecting a dichotomy in the impact of binding interface mutations. Whereas some specific mutations within interaction sites have been linked to oncogenic activity (Stehr et al., 2011), other binding site mutations are known to entirely disrupt critical interactions and overall protein function (Cho et al., 1994). Although we model the interaction sites of similar numbers of oncogenes and TSGs (238 and 246 respectively), we find that among the 50 genes with the highest enrichment of mutations within their interaction sites, the enrichment of oncogenes is 2.36-fold greater than the enrichment of TSGs. Nevertheless, PertInInt uncovers perturbed interaction interfaces in many genes that have been previously identified as drivers due to nonsense, frameshift, or other relatively disruptive mutations typically associated with TSGs (e.g., *RUNX1* and *FOXO1*). Indeed, enriched yet less common interaction-altering missense mutations uncovered by PertInInt may correspond to more subtle knockdown phenotypes or previously underappreciated oncogenic activities of genes traditionally characterized as TSGs.

### PertInInt Outperforms Previous Methods in Detecting Cancer Genes

Having demonstrated that PertInInt can identify interaction interfaces enriched in mutations across tumor samples, and that this is highly predictive of cancer genes, we next turn to assessing PertInInt’s performance as compared with previously published methods (Chang et al.,2016; Lawrence et al., 2014; Melloni et al., 2016; Mularoni et al., 2016; Porta-Pardo et al., 2015; Porta-Pardo and Godzik, 2014; Przytycki and Singh, 2017; Ryslik et al., 2013, 2014b; Tamborero et al., 2013; Ye et al., 2010) for detecting cancer driver genes (see STAR Methods). These methods differ substantially in terms of their statistical models and overall goals and, unlike PertInInt, are largely unable to distinguish among the various types of interaction and other functional perturbations affecting the identified genes.

**Figure 4.**
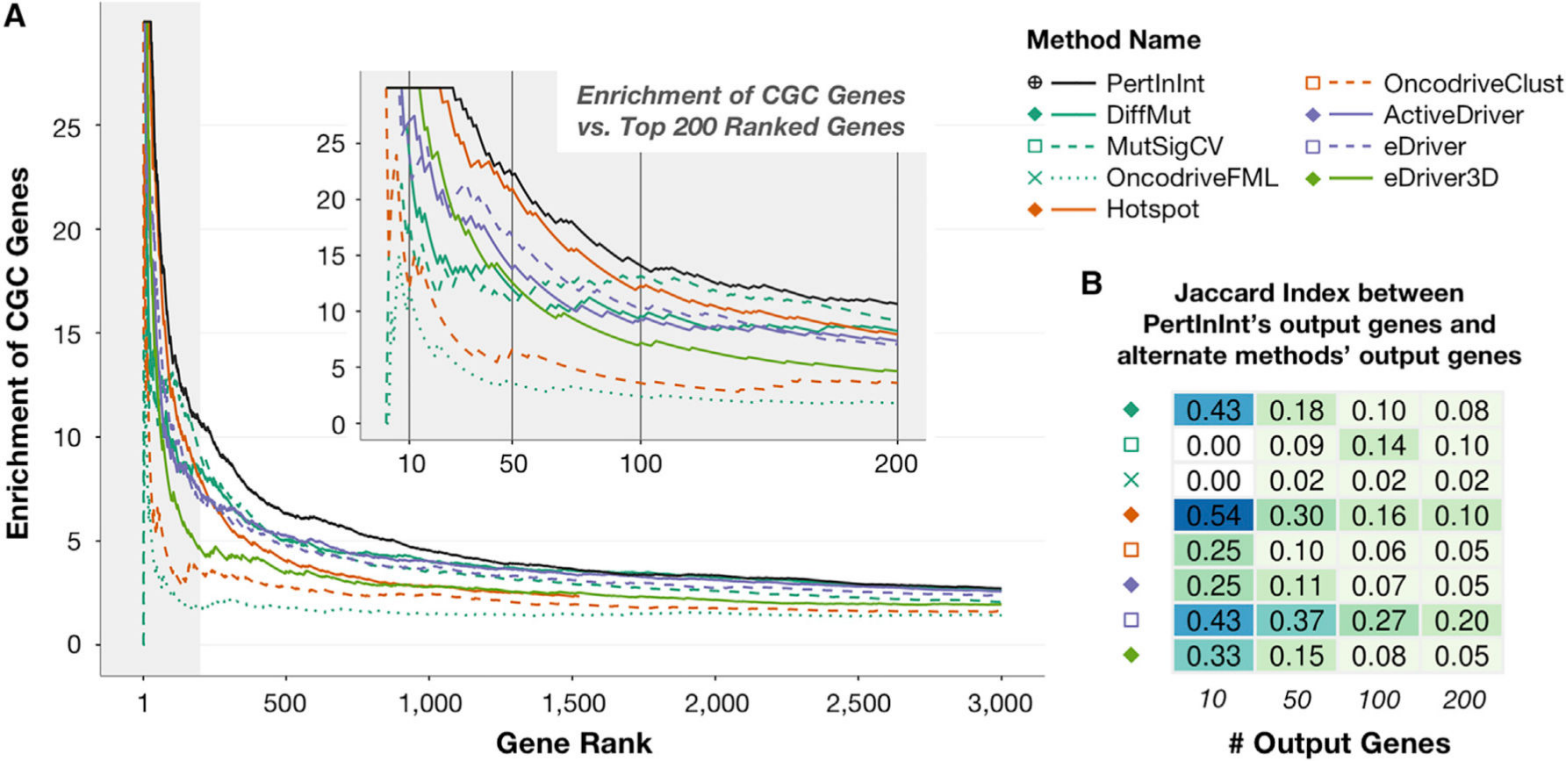
Detection of Known Cancer Genes from a Pan-Cancer Dataset by PertInInt and Alternate Methods Each driver gene detection method was run on the pan-cancer set of missense mutations. (A) Curves indicate the enrichment of CGC genes (y axis) as we consider an increasing number of output genes (x axis) for each driver gene detection method. Enrichment is computed as the ratio between the fraction of CGC genes in the set of top-scoring genes considered (i.e., the precision) and the fraction of CGC genes in the whole set of mutated genes (~0.0334). All methods scored at least 3,000 genes except for Hotspot (orange solid line), which only returned 1,530 genes and whose curve ends at that point. The gray shaded area highlights the plot to 200 genes, a closeup of which is shown in the inset. Vertical lines at 10, 50, 100, and 200 ranked genes in the inset correspond to gene set sizes featured in part (B). (B) JIs are calculated between the top 10, 50, 100, and 200 genes output by PertInInt and the corresponding top 10, 50, 100, and 200 genes output by each other method. Lighter colors indicate lower JIs and less overlap between the gene sets.

When applied to tumor samples from the pan-cancer dataset, our method has a greater enrichment for CGC genes than the other tested methods that we were able to run (Figure 4A). PertInInt also outperforms these other methods in terms of enrichment of CGC genes among top-ranked genes even after we exclude tumor samples from the six most highly mutated cancer types with 100+ missense mutations per patient on average, demonstrating that PertInInt’s superior pan-cancer performance is not driven by samples from cancer types that contribute large numbers of mutations (Figure S6). PertInInt’s greater enrichment as compared with other methods is observed as well for other lists of driver genes, and, furthermore, neither PertInInt nor any of the other tested methods show any enrichment for sets of genes that have been suggested to be unlikely to play roles in cancer (Figure S7; Table S2). Notably, the genes ranked highly by PertInInt differ substantially from those identified by other approaches (Figure 4B). Specifically, the set of genes identified by PertInInt has a consistently low Jaccard index (JI) with sets of genes ranked by alternate methods (JI < 0.5 across all methods for top 25 genes, JI < 0.25 across all methods for top 150 genes). Moreover, due to our analytical formulation, PertInInt can process the pan-cancer mutational data while considering multiple sources of data about protein functionality in 10 min on a single core of standard desktop; alternate methods each consider a limited set of mutational patterns and range in runtime from minutes to days (Table S3).

We also repeat our analysis on datasets restricted to samples from one cancer type, as many alternate methods that failed to run on the pan-cancer dataset are able to run on these substantially smaller subsets of tumor genomes. We find that in general across individual cancer datasets, PertInInt tends to achieve a higher area under the enrichment curve than other methods, including whole-gene methods, and a version of PertInInt that includes only subgene resolution tracks also outperforms other subgene methods (Figure S8). We note that since individual cancer types obviously have smaller total numbers of somatic mutations as compared with the combined pan-cancer dataset, the *Z* scores computed by PertInInt when run on individual cancers tend to be smaller than PertInInt’s pan-cancer *Z* scores; this trend is especially notable for cancer types with fewer samples and/or lower mutation rates. Similarly, fewer tracks can be evaluated for significance in the per-cancer analysis; this has a larger effect on interaction tracks in particular as they tend to involve fewer protein positions. Indeed, we find that the relative proportions of track types with positive *Z* scores for each cancer type are not notably different from each other, with the exception that the highly mutated cancer types (i.e., colorectal, lung, and stomach adenocarcinomas; skin cutaneous melanoma; and uterine corpus endometrial carcinoma, see Figure S3) each have a greater number of positively scoring domain and interaction tracks relative to other cancer types. Despite these differences when run on different cancer types, PertInInt is able to readily recover “cancer-specific” drivers (e.g., *EIF1AX* in uveal melanoma), and our analysis also reveals that genes that play dominant roles in certain cancer types may be important for smaller proportions of tumor samples in alternate cancer types as well (e.g., the same gene in thyroid carcinoma, Figure S9). Overall, our results show that PertInInt is a powerful method for evaluating mutational patterns across tumors of the same cancer type as well as across a pan-cancer dataset covering over 10,000 tumor genomes.

### Distinct Perturbed Molecular Mechanisms Uncovered across Genes

**Figure 5.**
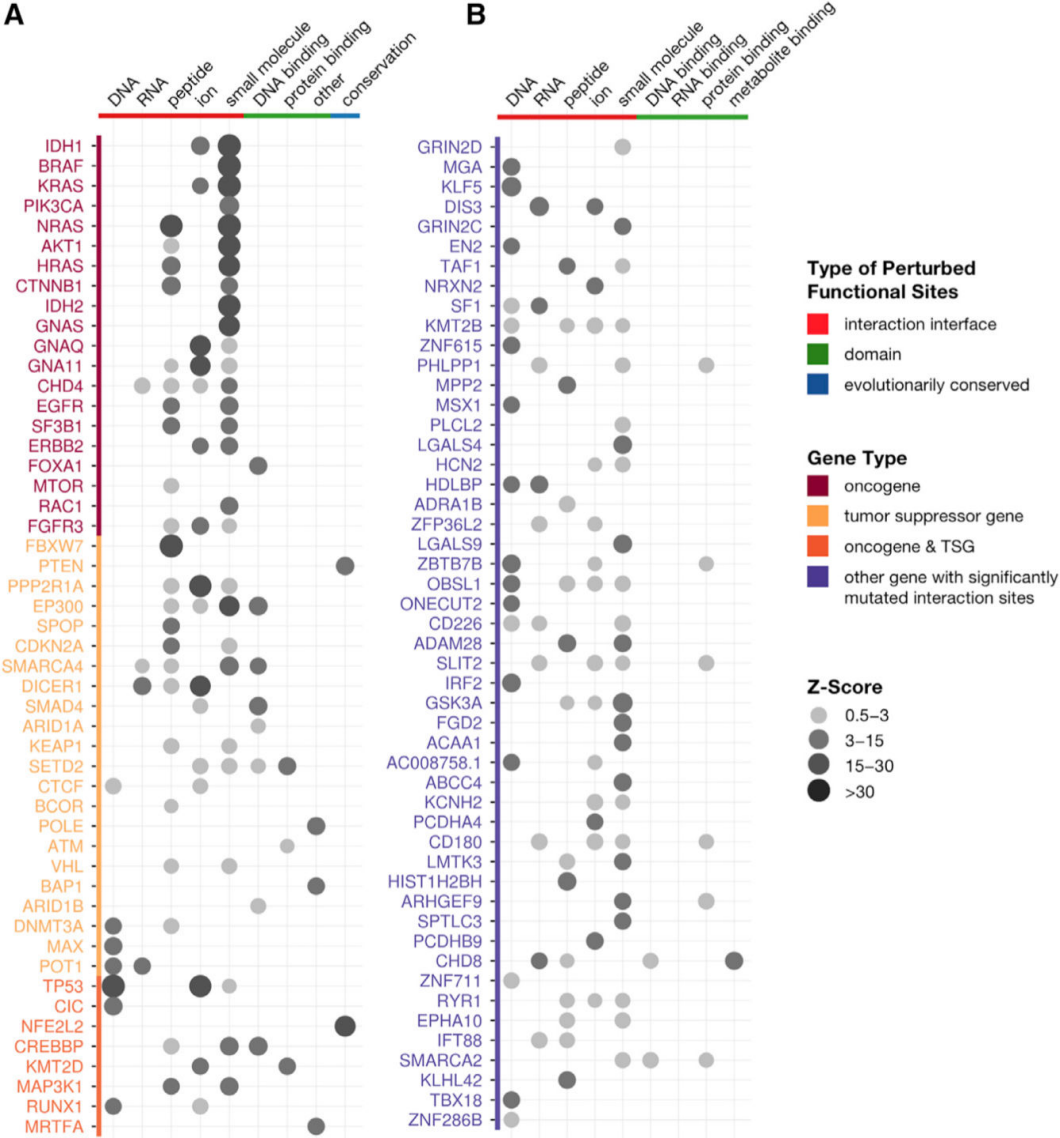
Perturbed Mechanisms across Oncogenes, Tumor Suppressor Genes, and Putative Cancer Genes Gene names are colored by driver status; genes that are not yet known to be cancer drivers but have a *Z* score ≥ 0.5 in one or more interaction tracks are in lavender. For each gene, the circles indicate the *Z* scores for enrichment of mutations in particular types of tracks, with interaction tracks in red, domain tracks in green, and the conservation track in blue. *Z* scores for mutational enrichments in domain tracks are shown only if the *Z* scores for the corresponding interaction tracks are < 0.5. *Z* scores for the conservation track are shown only if *Z* scores for all other track types are < 0.5. (A and B) (A) PertInInt’s top 50 ranked known cancer driver genes and (B) top 50 ranked putative cancer driver genes with a significantly mutated interaction track exhibit a wide range of perturbed functionalities.

Having shown that PertInInt is highly effective in identifying cancer genes, we next demonstrate its additional power to pinpoint which specific functional regions and mechanisms are perturbed by analyzing each track separately and determining which have positive *Z* scores (Table S4). Altogether, we find that 665 CGC genes have at least one subgene functionality track with a *Z* score ≥ 0.5, representing functional coverage of 93% of all CGC genes (Figure 5). Specifically, we find that DNA, RNA, peptide, ion, and small molecule interaction sites are enriched in mutations in 16%, 5%, 19%, 14%, and 22% of CGC genes, respectively; these numbers go up to 23%, 5%, 27%, and 24% of CGC genes if including those that are more broadly enriched in mutations across, respectively, DNA-binding, RNA-binding, peptide-binding, or metabolite-binding domains (as categorized in Pfam2Go; Mitchell et al., 2015). Up to 77% of CGC genes are enriched in mutations across at least one domain or interaction interface. We note that the perturbed nucleic acid and small molecule binding domains or interaction sites found across 45% of cancer genes would not be readily identified by analyses that focus exclusively on protein–protein interaction alterations (Porta-Pardo et al., 2015).

**Figure 6.**
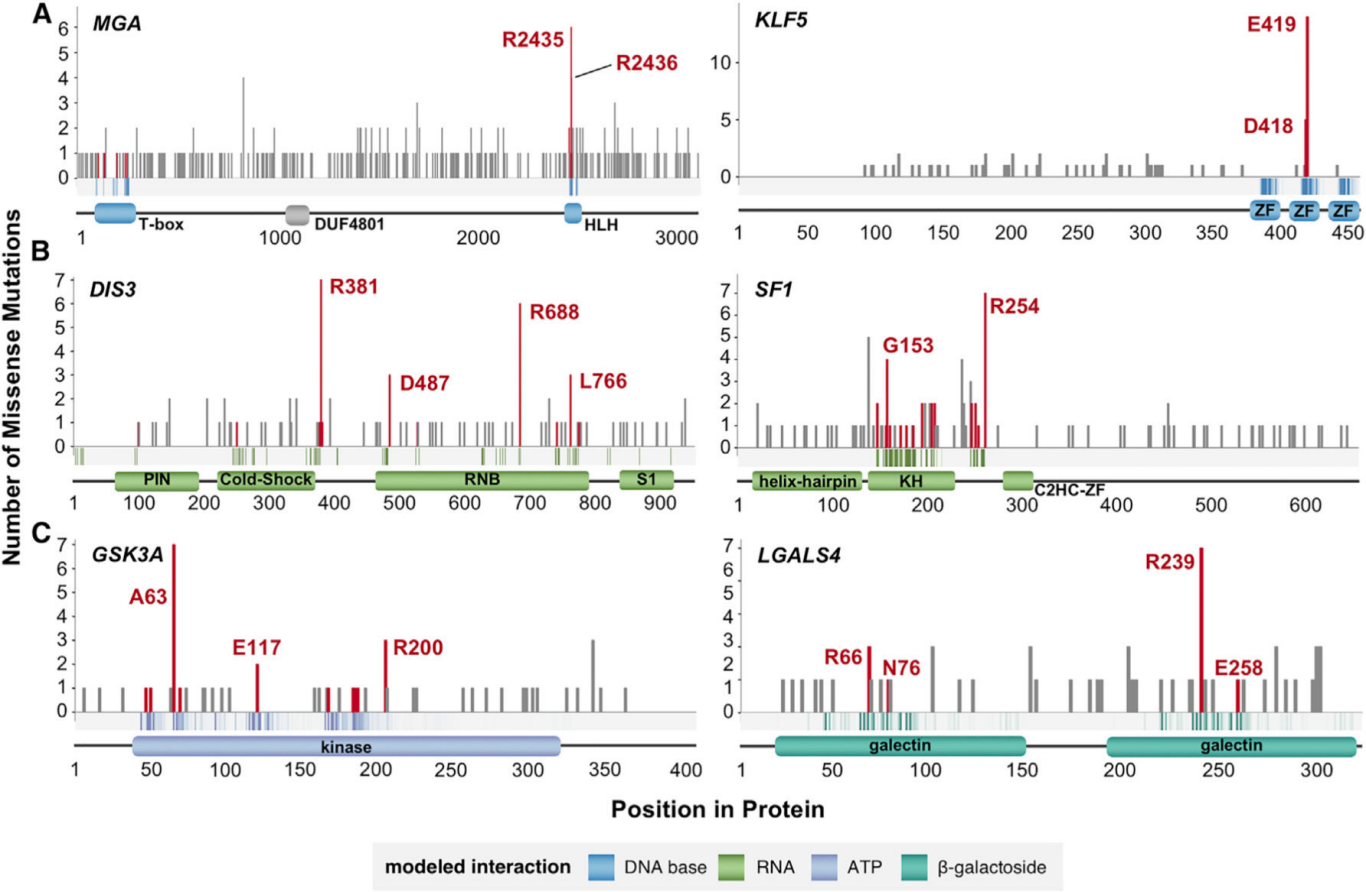
Examples of Genes Ranked Highly by PertInInt that Are Not Known to Be Drivers Across the length of each gene (x axis), the number of missense mutations at each protein position is given (y axis). Vertical bars corresponding to mutations affecting binding sites are colored red. The band along the x axis depicts the likelihoods with which residues at each protein position are expected to interact with the specified ligand, with darker bars corresponding to higher (≥ 0.25) binding likelihoods. Domain locations and names are shown below. (A) Putative cancer genes *MGA* and *KLF5* are enriched for mutations in DNA base-binding positions. (B) Putative cancer genes *DIS3* and *SF1* are enriched for mutations in RNA-binding positions. (C) Putative cancer genes *GSK3A* and *LGALS4* are enriched for mutations in small molecule- (ATP and β-galactoside sugar, respectively) binding positions.

We now highlight a few of these genes that, though not present in the CGC, were uncovered by PertInInt as having significantly mutated interaction interfaces. For instance, transcription factors *MGA* and *KLF5* harbor mutations within their basic helix-loop-helix and C2H2-ZF domains, respectively, that alter their DNA base-binding positions (Figure 6A), suggesting cancer-specific changes to normal DNA-binding and downstream regulatory activity. Indeed, *KLF5*’s E419Qmutation has recently been experimentally shown to change wild-type binding preferences and increase the expression of tumor progression genes *in vivo* (Zhang et al., 2018). Similarly, *MGA* normally subdues the activity of well-known oncogene *MYC*; its frequent deletion, truncation, or mutated binding properties across cancers further indicates its role as a tumor suppressor (Schaub et al., 2018). We also find that two RNA-binding genes *DIS3* and *SF1* exhibit significant mutations in their putative RNA-binding sites, with recurrent mutations in *DIS3* altering multiple distinct RNA-contacting positions (Figure 6B). In support of our predictions, *DIS3* is recurrently mutated in blood and skin cancers and has been identified as a candidate oncogene in colorectal cancer (de Groen et al., 2014). *SF1* is recurrently mutated across cancers in a mutually exclusive fashion (i.e., indicating its analogous functionality) to *RBM10*, a gene found to drive aberrant splicing events in cancer (Seiler et al., 2018).

PertInInt also newly implicates a number of genes—present neither in the CGC nor on other lists of known cancer genes (Bailey et al., 2018; Kandoth et al., 2013; Lawrence et al., 2014; Vogelstein et al., 2013)—with mutations that appear to alter critical small molecule interaction sites (Figure 6C). The highly conserved kinase *GSK3A* for instance harbors a significant enrichment of mutations altering its ATP-binding positions. Supporting our prediction, suppression of this gene is associated with impaired growth and induction of apoptosis, and it has recently been proposed as a potential therapeutic target in acute myeloid leukemia (Banerji et al., 2012; McCubrey et al., 2014). We also find that the S-type lectin *LGALS4* has an enrichment of mutations altering the β-galactoside sugar-binding positions in its galectin domains; indeed, *LGALS4* has been linked to the regulation of the cancer-relevant Wnt signaling pathway and has been experimentally implicated as a tumor suppressor in colorectal cancer cells *in vitro* (Satelli et al., 2011).

To more broadly characterize PertInInt’s novel cancer gene predictions, we use gene set enrichment analysis (GSEA) (Mootha et al., 2003; Subramanian et al., 2005) with Reactome pathways (Jassal et al., 2020) on the ranked list of non-CGC genes output by PertInInt with combined score ≥ 1 (Table S5). GSEA uncovers 12 enriched Reactome pathways at false discovery rate (FDR) corrected p value (or *q*-value) < 0.2, including known cancer pathways such as *Ras* (*q*-value = 0.102), and signaling pathways mediated by known cancer genes, such as *CREB1* (*q*-value = 0.042), *NOTCH3* (*q*-value = 0.071), and *PDGF* (*q*-value = 0.195). In addition, a pathway related to cell adhesion, which is known to be disrupted especially in metastatic tumors, is also enriched (e.g., L1-ankyrin interactions, *q*-value = 0.078). Intriguingly, two enriched pathways suggest that nervous system-related functionalities may play roles across tumors (*q*-value < 0.2), an observation that has only very recently been explored (Zeng et al., 2019).

## DISCUSSION

In this work, we have introduced a fast, integrative framework to detect cancer driver genes by uncovering whether somatic mutations across tumors are enriched in sites of different types of functionalities. Our method utilizing this framework, PertInInt, integrates knowledge from the largest set of protein–ligand interaction sites to date (Ghersi and Singh, 2014; Kobren and Singh, 2019) with additional biological data regarding subgene functionality and whole gene mutability (Figures 1 and S1). When applied to over 10,000 tumor samples from 33 cancer types, PertInInt reveals a broad range of perturbed functionalities in several known driver genes as well as in relatively rarely mutated genes with predicted tumorigenic roles (Figures 3A, 3B, and 5). Notably, PertInInt finds that mutations within many known driver genes are enriched in protein interaction interfaces (Figure 2A) and more broadly implicates interaction perturbation as a frequent phenomenon in cancer cells (Figure 5).

Analyses of predicted cancer-relevant coding mutations often involve—whenever possible—assessing their putative effect with respect to protein structure (Bailey et al., 2018; Chang et al., 2016; Kamburov et al., 2015; Niknafs et al., 2013; Raimondi et al., 2017). Although using structure directly to identify relevant mutations is rarely scalable in terms of runtime and coverage (Ryslik et al., 2013), PertInInt’s use of structurally predefined regions mediating protein interactions makes large-scale analyses in the context of protein structure feasible. Moreover, since cancer-driving genetic aberrations do not always involve mutation of protein–ligand interaction interfaces, a critical additional feature of PertInInt—that extends its coverage to all human genes—is that it seamlessly incorporates additional lines of evidence regarding protein site functionality. While here we have demonstrated that PertInInt effectively utilizes per-site evolutionary conservation and domain knowledge, we anticipate that encoding more sources of functional information within our framework (e.g., known phosphorylation sites or intrinsically disordered regions) will unearth other driver mutations and alternate mechanisms of action. Incorporating structurally resolved information from protein–protein interaction networks will also be a valuable direction for future work.

Genes that are frequently mutated across their lengths tend not to overlap genes that exhibit nonrandom patterns of mutations across individual protein positions, a pattern that has previously been leveraged to distinguish TSGs from oncogenes (Tokheim et al., 2016b; Vogelstein et al., 2013). By incorporating whole gene mutability information into our existing framework, we are able to uncover and profile a much more comprehensive set of both oncogenes and TSGs (Figures 2B and 3C). Although previous methods have also considered the frequency and spatial patterning of mutations within genes together (Korthauer and Kendziorski, 2015; Lawrence et al., 2013; Tokheim et al., 2016b), we also simultaneously infer specific perturbed molecular mechanisms within uncovered genes. We note that while mutation deleteriousness predictors—developed both in the context of cancer (Carter et al., 2009; Shihab et al., 2013) and otherwise (Adzhubei et al., 2010)—can evaluate the impact of somatic mutations, they tend to integrate multiple sources of protein site functionality information via complex statistical or machine learning approaches, where the contribution of each data source and thus subsequent mechanistic interpretations are obscured. In contrast, by determining mutational enrichments in specific types of functional sites, PertInInt is able not only to identify cancer-relevant genes but also to begin to explicitly reason about the biomolecular impacts of mutations. Indeed, uncovering the mechanisms of action for cancer-driving mutational events has been a major bottleneck in the critical step of translating this knowledge to improve patient care and outcomes (The ICGC/TCGA Pan-Cancer Analysis of Whole Genomes Consortium et al., 2020).

Given the success of large-scale cancer genome sequencing consortia projects in expanding our knowledge of basic cancer biology (Bailey et al., 2018; Ding et al., 2018; Hoadley et al., 2018; Sanchez-Vega et al., 2018), coupled with the decreased cost of genome sequencing, it is clear that sequencing tumor genomes will be routine practice in both basic science and clinical settings, thereby rapidly increasing the number of sequenced tumors available for analysis. Importantly, PertInInt’s analytical framework enables it to efficiently process increasing numbers of tumor genomes; further, this speed is accompanied by better identification of cancer-relevant genes when run on larger numbers of tumor samples (Figure S10). Since PertInInt’s underlying analytical framework is general, we anticipate that it will also be effective in other settings. For example, because very few non-coding somatic mutations in cancer tend to be recurrent (Khurana et al., 2016), it may be especially powerful for identifying regulatory regions with an enrichment of mutations within sites associated with different measures of functionality (e.g., binding sites for different proteins).

In the future, one of the most tantalizing prospects of cancer genomics is its potential in transforming clinical practice. While identifying and linking cancer mutations to personalized treatments remains a daunting challenge, PertInInt dramatically accelerates the detection of rare mutational driver events from sequenced tumors while providing important information about their mechanisms of action, a key step in developing and customizing targeted therapeutic regimens.

## STAR★METHODS

### RESOURCE AVAILABILITY

#### Lead Contact

Further information and requests for resources should be directed to and will be fulfilled by the lead contact, Mona Singh (mona@cs.princeton.edu).

#### Materials Availability

This study did not generate new unique reagents or materials.

#### Data and Code Availability

All original code generated during this study are available at http://github.com/Singh-Lab/PertInInt.

### METHOD DETAILS

#### Protein Site-Based Functional Tracks

Any pre-defined functional region of a protein can be encoded as a track in the PertInInt framework. Currently, we consider three types of per-site functional annotations—interaction, domain, and conservation—the former two of which may yield multiple subgene resolution tracks per protein. Each type of track is described in more detail below.

##### Interaction Tracks

Interaction tracks correspond to portions of a protein that are inferred to interact with ligands. These tracks arise in two ways.

First, we leverage sequence homology directly to transfer information from co-complex structures to human protein sequences as previously described (Ghersi and Singh, 2014). For proteins with one or more regions whose structure in complex with a ligand could be homology-modeled, we introduce a track for each contiguous homology-matched region. Per-position weights reflect the observed residue-to-ligand proximities, computed as the fraction of atoms in the amino acid residue found within 4.0Å of the ligand.

Second, we utilize the set of “confident” domain–ligand interactions from the InteracDome database (v0.3) (Kobren and Singh, 2019) to identify putative ligand-binding positions. We subset this collection to the 9,142 domain–ligand interactions across 1,850 domains that were characterized by InteracDome using at least five structural instances. Each position within an InteracDome domain is associated with a “binding frequency” between 0 and 1 that corresponds to the fraction of the time residues in this position were found to be in contact with the ligand of interest when analyzing co-complex structures. For each human protein, we identify instances of InteracDome domains using HMMER (v2.3.2 and v3.1b2), and require complete, high-scoring domain instances as previously described (Eddy, 2011; Finn et al., 2014; Kobren and Singh, 2019). Within a protein, there is a separate track for each domain–ligand instance within it; this track consists of the residues comprising the match states of the domain, and the weights of these residues are the binding frequencies for the ligand in the corresponding domain positions.

Finally, we note that some domain interactions are mediated not by individual domain instances but by repeating instances of the same domain family. To capture these interfaces, we also consider additional tracks encoding multiple instances of the same domain family in a protein; these “aggregate” tracks span noncontiguous intervals that correspond to the locations of individual domain instances, with track positions weighted according to the binding frequencies at corresponding domain match states as described above. Interaction domain tracks corresponding to domain families with 40+ instances in the same protein are replaced by their aggregate tracks.

##### Domain Tracks

For each Pfam-A (v31.0) domain instance within a protein sequence, there is a domain track that specifies which amino acids comprise the domain (Finn et al., 2014). Domain tracks span the length of the protein, and positions within and outside of the domain instance are respectively assigned weights of 1 and 0. We again also encode aggregate domain tracks as before to model functional regions mediated by repetitive domain families.

##### Conservation Tracks

Each protein has a single conservation track. We obtain the 100-vertebrate cross-species protein multiple sequence alignment from the UCSC Genome Browser (Meyer et al., 2013) and compute per-protein-position conservation-based functionality weights by multiplying the fraction of non-gap residues in the column by the Jensen-Shannon divergence (JSD) between those non-gap residues and a Blosum 62 background amino acid distribution (Capra and Singh, 2007).

#### Per-track Functional Mutation Scores

Suppose we have a protein sequence of length *L* spanning positions *P* = {*p*_1_,...,*p*_*L*_}. This protein is associated with multiple “tracks” *W*, each defined as *W*⊆*P*, where each position *p*_*i*_∈*W* is associated with a real-valued weight *w*_*i*_∈[0, 1] reflecting its functionality with respect to the track. Suppose there are *n* cancer somatic missense mutations across a cohort of tumor samples that fall in positions included in track *W*. For each mutation *i*, let *z*_*i*_∈{*z*_1_, …, *z*_*n*_} be the weight in track *W* of the position where that mutation lies. We further consider the case where each mutation *i* is associated with a value *f*_*i*_∈(0, 1]; here, each *f*_*i*_ is set to the proportion of sequencing reads that contain the mutation (i.e., its subclonal fraction), which has previously been shown to be associated with a mutation’s relevance in cancer (McGranahan et al., 2015). The score of the somatic mutations with respect to track *W* is then defined as:

(Equation 1) $$S_{W}=\sum_{i=1}^{n}f_{i}z_{i}.$$

Intuitively, this score reflects the extent to which somatic mutations are falling into functionally important positions within a track.

#### Per-track Analytical *Z* score Calculations

For a given score *S*_*W*_ for a track, we next want to determine if this score is higher than we would expect by chance. One approach would be to repeatedly randomize the mutations within the positions of the track and use the distribution of resulting scores to compute an empirical p value. Here we show that we can determine the significance of these scores analytically, obviating the need for empirical mutation shuffles and dramatically improving runtime (Figure S2). Note that in the absence of any selective pressure, the values *z*_1_, …, *z*_*n*_ are independent and identically distributed (i.i.d.) random variables. We leverage this observation to directly compute the significance of *S*_*W*_. First, we model all mutation locations *z*_*i*_ as being drawn from the same background mutation model *λ*_1_, …, *λ*_*L*_, where *λ*_*i*_ is the probability that a mutation affects position *i*. If every position *i* within a protein of length *L* is equally likely to harbor a missense mutation, *λ*_*i*_ = 1/*L*. Here, we incorporate codon-specific missense mutation probabilities as well as cancer-specific C/G-mutation biases into our background mutation model (see “Per-site background mutational model” STAR Methods section below). We linearly scale these values with respect to each track *W* such that $\sum_{j\in W}\lambda_{j}^{W}=1$. The expected weight of the position in which mutation *i* lies $\left(E\left[z_{i}\right]\right)$ and its variance $\left(\sigma_{z_{i}}^{2}\right)$ with respect to this null distribution are computed as

$$E\left[z_{i}\right]=\sum_{j\in W}\lambda_{j}^{W}w_{j}$$

and

$$\sigma_{z_{i}}^{2}=E\left[z_{i}^{2}\right]-{\left(E\left[z_{i}\right]\right)}^{2}=\sum_{j\in W}\lambda_{j}^{W}w_{j}^{2}-{\left(\sum_{j\in W}\lambda_{j}^{W}w_{j}\right)}^{2}.$$

Because the total score of the set of mutations affecting track *W* (i.e., *S*_*W*_) is a sum of independent random variables (Equation 1), the expectation and variance of *S*_*W*_ can also be calculated directly as

(Equation 2) $$E\left[S_{W}\right]\;=\;\sum_{i=1}^{n}E\left[f_{i}z_{i}\right]\;\;=\;\sum_{i=1}^{n}f_{i}\cdot E\left[z_{i}\right]\;\;=\;\sum_{i=1}^{n}f_{i}\left(\sum_{j\in W}\lambda_{j}^{W}w_{j}\right)$$

and

(Equation 3) $$\sigma_{S_{W}}^{2}\;=\;\sum_{i=1}^{n}\sigma_{f_{i}z_{i}}^{2}\;\;=\;\sum_{i=1}^{n}f_{i}^{2}\sigma_{z_{i}}^{2}\;\;=\;\sum_{i=1}^{n}f_{i}^{2}\left(\sum_{j\in W}\lambda_{j}^{W}w_{j}^{2}-{\left(\sum_{j\in W}\lambda_{j}^{W}w_{j}\right)}^{2}\right).$$

Finally, to determine the significance of the actual score *S*_*W*_, which indicates the propensity of somatic mutations to fall into highly weighted positions in a track, since the sum of independent random variables tends towards a normal distribution, we compute the mutational enrichment *Z* score for each track *W* as

(Equation 4) $$Z_{W}=\frac{S_{W}-E\left[S_{W}\right]}{\sigma_{S_{W}}}.$$

We note that if we (1) restricted each weight within a track to be 0/1 rather than real-valued, (2) restricted mutations to have equal *f*_*i*_ values of 1, and (3) restricted the *λ*_*i*_ to be uniform across the track, we could determine per-track significance analytically using the binomial distribution. Note that with these restrictions, however, we would not be able to incorporate real-valued functionality weights from conservation or interaction tracks, subclonal mutation fractions, or mutational signatures.

#### Per-site Background Mutational Model

We model the likelihoods of protein positions *p*_1_, …, *p*_*L*_ to harbor a missense mutation as *λ*_1_, …, *λ*_*L*_ such that

(Equation 5) $$\lambda_{j}=\sum_{d\in \{1,2,3\}}\left(B_{jd}\cdot \sum_{u\in \{A,T,C,G\}}M_{jdu}\right)$$

where

$$B_{jd}=\left\{ \begin{array}{ll} 1 & \text{if}\;\text{the}\;d\text{th}\;\text{nucleotide}\;\text{in}\;\text{the}\;\text{codon}\;\text{at}\;\text{position}\;p_{j}\;\text{is}\;\text{A}\;\text{or}\;T\\ b & \text{otherwise},\;\text{where}\;b\;\text{is}\;\text{the}\;\text{relative}\;\text{frequency}\;\text{of}\;a\;\text{C/G}\;\text{mutation}\;\text{in}\;\text{the}\;\text{pan-cancer}\;\text{dataset}\;\text{as}\;\text{compared}\\ & \text{to}\;\text{a}\;\text{A/T}\;\text{mutation}\;(\text{i.e.},\;3.063) \end{array}\right.$$

and

$$M_{jdu}=\{\begin{array}{ll} 1 & \text{if}\;\text{changing}\;\text{the}\;d\text{th}\;\text{nucleotide}\;\text{in}\;\text{the}\;\text{codon}\;\text{at}\;\text{position}\;p_{j}\;\text{to}\;u\;\text{results}\;\text{in}\;\text{a}\;\text{missense}\;\text{mutation}\\ 0 & \text{otherwise} \end{array}$$

#### Between-Track Analytical Covariance Calculation

In our framework, a single protein may be associated with *multiple* tracks, each representing a distinct aspect of protein functioning. Since tracks can share positions, the track scores with respect to a set of somatic mutations are not independent of each other, and thus we need to determine their covariance.

Suppose we consider two tracks *V*⊆*P* and *W*⊆*P*, where each position *p*_*i*_∈*V* is associated with a weight *v*_*i*_ and each position *p*_*i*_∈*W* is associated with a weight *w*_*i*_. Suppose there are *m* mutations (with associated values $f_{1}^{\prime },\ldots ,f_{m}^{\prime }$) that involve positions within track *V*, and *n* mutations (as before with associated values *f*_1_, …, *f*_*n*_) that involve positions within track *W*. Let *y*_1_, *y*_2_, …, *y*_*m*_ be the weights of the positions that the *m* mutations in track *V* fall into, and let *z*_1_, *z*_2_, …, *z*_*n*_ be the weights of the positions that the *n* mutations in track *W* fall into. Scores are thus calculated as before for tracks *V* and *W* as

$$S_{V}=\sum_{i=1}^{m}f_{i}^{\prime }y_{i}$$

and

$$S_{W}=\sum_{i=1}^{n}f_{i}z_{i}.$$

Let *X* = *V*∩*W*. If the two tracks do not overlap (i.e., *X* = ∅), then the covariance between *S*_*V*_ and *S*_*W*_ is 0. Otherwise, note that $S_{V}=S_{V}^{\prime }+S_{V}^{X}$, where $S_{V}^{\prime }=\sum_{j\notin X}f_{j}^{\prime }y_{j}$ and $S_{V}^{X}=\sum_{j\in X}f_{j}^{\prime }y_{j}$. Similarly, $S_{W}=S_{W}^{\prime }+S_{W}^{X}$. Therefore, we can write covariance as

$$\mathrm{cov}\left[S_{V},S_{W}\right]=\mathrm{cov}\left[\left(S_{V}^{\prime }+S_{V}^{X}\right),\left(S_{W}^{\prime }+S_{W}^{X}\right)\right].$$

Because the covariance is bilinear, we can now expand this equation as

$$\mathrm{cov}\left[\left(S_{V}^{\prime }+S_{V}^{X}\right),\left(S_{W}^{\prime }+S_{W}^{X}\right)\right]=\mathrm{cov}\left[S_{V}^{\prime },S_{W}^{\prime }\right]+\mathrm{cov}\left[S_{V}^{\prime },S_{W}^{X}\right]+\mathrm{cov}\left[S_{V}^{X},S_{W}^{\prime }\right]+\mathrm{cov}\left[S_{V}^{X},S_{W}^{X}\right].$$

Finally, because mutations landing in track *V* outside of the overlap region *X* have no bearing on *S*_*W*_ and vice versa, the first three covariance terms in the equation above will be evaluated as 0, leaving us with

$$\mathrm{cov}\left[S_{V},S_{W}\right]=\mathrm{cov}\left[S_{V}^{X},S_{W}^{X}\right].$$

In our framework, we compute covariance conditional on the *q* mutations observed to fall on positions shared by tracks *V* and *W*. Let $F=f_{i_{1}}^{2}+\cdots +f_{i_{q}}^{2}$, with the *f*_*i*_ associated with the *q* mutations in *X*. With the number of mutations *q* fixed, we have

$$\mathrm{cov}\left[S_{V}^{X},S_{W}^{X}\right]=\mathrm{cov}\left[\sum_{j=1}^{q}f_{i_{j}}y_{i_{j}},\sum_{j=1}^{q}f_{i_{j}}z_{i_{j}}\right]=\sum_{j=1}^{q}\sum_{k=1}^{q}\mathrm{cov}\left[f_{i_{j}}y_{i_{j}},f_{i_{k}}z_{i_{k}}\right].$$

Note that the *same* mutations from tracks $S_{V}^{X}$ and $S_{W}^{X}$ land on the *same* position in the overlap region and simultaneously impact the *S*_*V*_ and *S*_*W*_ scores, whereas any other pair of mutations *j* ≠ *k* are independent. Hence,

(Equation 6) $$\mathrm{cov}\left[S_{V}^{X},S_{W}^{X}\right]\;=\;\sum_{j=1}^{q}\mathrm{cov}\left[f_{i_{j}}y_{i_{j}},f_{i_{j}}z_{i_{j}}\right]\;\;=\;\sum_{j=1}^{q}f_{i_{j}}^{2}\cdot \mathrm{cov}\left[y_{i_{j}},z_{i_{j}}\right]\;\;=\;F\cdot \mathrm{cov}\left[y_{i_{1}},z_{i_{1}}\right]\;\;=\;F\left(\sum_{j\in X}\lambda_{j}^{X}v_{j}w_{j}-\left(\sum_{j\in X}\lambda_{j}^{X}v_{j}\right)\left(\sum_{j\in X}\lambda_{j}^{X}w_{j}\right)\right)$$

##### Analytical Formulation Enables Precomputation

Note that the per-track expectation, variance and covariance calculations (Equations 2, 3, and 6) can each be rewritten as $C\cdot \sum_{i}f_{i}$ or $C\cdot \sum_{i}f_{i}^{2}$, where *C* is fixed per track. We therefore precompute the per-track expectations, variances, and cross-track covariances assuming a single mutation of value 1, and scale these precomputed values at runtime by the mutations observed to fall into each track; this allows PertInInt to achieve an additional 16–18× speedup at runtime (Figure S11).

#### Whole Gene Mutability Tracks

Using the same analytical formulation described above, we can also compute a *Z* score per gene reflecting whether the gene is more mutated overall than we might expect. We define a natural variation track of length *L* = 19,460 for each gene, where the entry corresponding to the gene of interest has a functionality weight of 1, and all other entries have weights of 0 (i.e., one-hot gene encodings). We then compute a corresponding background mutability probability distribution *λ*_1_,…*λ*_*L*_ based on how much each gene varies naturally across healthy human populations. Specifically, for each of 2,504 individuals included in the 1000 Genomes Project (The 1000 Genomes Project Consortium, 2012), we first min-rank all protein-coding genes by their variant count, linearly scale these ranks to fall between 0 and 1, then round each normalized rank down to its nearest hundredth, which we refer to as its bin. We compute the expected bin value (across individuals) for each gene, and finally to derive the values of *λ*_1_,…, *λ*_*L*_ linearly scale these per-gene expected bin values such that they sum to 1 across all genes. For each track, we use this background mutation model and the *n* mutations observed to fall across all 19,460 genes to analytically compute *per-gene* expectations, variances, and *Z* scores as before. Note that PertInInt models 23,278 genes—of which 20,356 are on chromosomes 1–22, X or Y—but only 19,460 genes were profiled in the 1000 Genomes Project, and thus only this many genes have associated natural variation tracks. The covariance between the natural variation track and all subgene tracks is set to 0.

Since the whole-gene track *W* for gene *G*_*j*_ is a one-hot encoding, we can simplify Equation 1 as $S_{W}=\sum_{i\in G_{j}}f_{i}$, Equation 2 as $E\left[S_{W}\right]=\lambda_{j}^{W}\sum_{i=1}^{n}f_{i}$ and Equation 3 as $\sigma_{S_{W}}^{2}=\lambda_{j}^{W}\left(1-\lambda_{j}^{W}\right)\sum_{i=1}^{n}f_{i}^{2}$.

Because the number of mutations affecting all genes is often substantially larger than the number of mutations to affect any single gene, the whole-gene *Z* scores can be much larger than for the other tracks. We thus effectively subsample the total number of mutations by a factor *s*—set to $\frac{1}{\sqrt{n}}$ in our implementation—to compute the whole-gene *Z* scores using the values below before combining them with other subgene *Z* scores:

$$S_{W}=s\sum_{i\in G_{j}}f_{i},$$

$$E\left[S_{W}\right]=s\lambda_{j}^{W}\sum_{i=1}^{n}f_{i},$$

$$\sigma_{S_{W}}^{2}=s\lambda_{j}^{W}\left(1-s\lambda_{j}^{W}\right)\sum_{i=1}^{n}f_{i}^{2}.$$

#### Combining Per-track *Z* scores for Each Protein

We evaluate the significance of the scores for all tracks simultaneously using a multivariate normal distribution. Recall that our per-track somatic mutation functional scores (*S*_*W*_, Equation 1) and their analytically-derived *Z* scores (Equation 4) computed for random assignments of mutations are normally distributed when the number of mutations (*n*) is sufficiently large (i.e., by the Central Limit Theorem).

For each track *W*, we empirically determine this minimum *n* by randomly assigning up to 500 mutations to the track 1,000 times in accordance with the corresponding background mutation model (i.e., the *λ*_*i*_’s) and recomputing *S*_*W*_ each time. At each mutation count, we ask whether we can reject the null hypothesis that the mutation functional scores are normally distributed via the Shapiro-Wilk test with p value < 5e-5. We keep track of the minimum number of mutations per track where we could no longer confidently reject the normality assumption. Only scores derived from mutated tracks with the corresponding required minimum mutation count are modeled together in our multivariate Gaussian. We pre-compute this minimum mutation count value for each track (i.e., before evaluating any cancer somatic mutation data).

For each mutated protein, we compute a single combined score using a weighted *Z*-transform test with correlation correction (Zaykin, 2011) as

(Equation 7) $$Z=\frac{\sum_{i=1}^{k}c_{i}Z_{i}}{\sqrt{\sum_{i=1}^{k}c_{i}^{2}+2\sum_{i<j}c_{i}c_{j}r_{ij}}}$$

where *k* is the number of tracks with their required minimum mutation count and positive *Z* scores, *Z*_*i*_ corresponds to the *Z* score associated with track *i*, *c*_*i*_ is a weight indicating the “confidence” of track *i*, and *r*_*ij*_ is the correlation between tracks *i* and *j* (i.e., *r*_*ij*_ = cov(*S*_*i*_, *S*_*j*_)/*σ*_*i*_*σ*_*j*_ ). In order to consider each type of functionality data equally, we assign per-track confidences *c*_*i*_ such that the four functional track groups (i.e., interaction, domain, conservation, and natural variation; Figures 1A and S1) each contribute a quarter of the overall confidence. Within the interaction and domain track groups, where there may be 2+ tracks per group, confidence weights are assigned proportionally to $\sqrt{m}$, where *m* is the total number of mutations to fall into positively-weighted positions in the track. Finally, we assign a single score per gene by taking the maximum combined score achieved by any of its corresponding protein isoforms.

### QUANTIFICATION AND STATISTICAL ANALYSIS

#### Cancer Mutation Data Preparation

We downloaded all open-access TCGA somatic exome mutation data and RNA-seq expression data from NCI’s Genomic Data Commons on July 15, 2017 (Fan et al., 2016; Grossman et al., 2016). We convert gene expression values (FPKM) to transcripts per million (TPM) and exclude mutations from genes that were expressed at <0.1 TPM in the corresponding tumor sample. For the 765 samples with missing expression data, we exclude mutations from genes that were expressed at <0.1 TPM on average across other tumor samples of the same tissue type. These steps resulted in a filtered set of 1,141,609 missense, 442,070 silent, and 94,813 nonsense mutations across 18,613 genes from 33 cancer types (Figure S3); note that we consider the unfiltered (by expression) set of 1,473,729 missense, 578,407 silent and 118,921 nonsense mutations across 19,550 genes when running alternate methods and when running PertInInt to compare to alternate methods. We combine COAD and READ cancer types into the COAD-READ group, and GBM and LGG cancer types into the GBMLGG group for per-cancer performance testing (Figures S8 and S9).

#### Runtime Analysis

PertInInt, as well as all algorithm variants of PertInInt and all alternate methods, are run as sole processes on single CPUs, each with a 2.4–2.7 Ghz processor and 30GB of RAM. Methods are timed using Python’s time package, and the real (i.e., “wall clock”) elapsed time is reported.

#### Testing Related Driver Detection Methods

We classify alternate cancer driver detection methods based on the mutational patterns they detect; these include whole gene enrichment, *de novo* linear clustering, enrichment in linear externally defined regions, *de novo* three-dimensional (3D) clustering, or enrichment in 3D externally defined regions (as in Porta-Pardo et al., 2017). We include methods from each of these five groups that require only mutational and/or structural input from the user and have open-source implementations that run locally on a 64-bit Linux machine using sample input. We test the whole gene methods DiffMut (Przytycki and Singh, 2017), MutSigCV (Lawrence et al., 2014), and OncodriveFML (Mularoni et al., 2016); the linear clustering methods Hotspot (Chang et al., 2016), OncodriveClust (Tamborero et al., 2013), and NMC (Ye et al., 2010); the linear externally defined regions methods ActiveDriver (Reimand and Bader, 2013), eDriver (Porta-Pardo and Godzik, 2014), and LowMACA (Melloni et al., 2016); the 3D clustering methods GraphPAC (Ryslik et al., 2014b), iPAC (Ryslik et al., 2013), and SpacePAC (Ryslik et al., 2014a); and the 3D externally defined regions method eDriver3D (Porta-Pardo et al., 2015). We note that in addition to overall mutation frequency, MutSigCV also considers linear clustering of mutations within genes and the functional impact of mutations based on evolutionary conservation.

All methods including PertInInt are run on the same mutation datasets before our filtering step of limiting to mutations from expressed genes. Additional data files required for individual methods are obtained from their most recent online repositories or otherwise from their original publications. For 3D clustering methods, we select a single structural template for each human protein wherever possible as suggested (i.e., preferring native over bound form, longer length, higher sequence identity, higher resolution, and smaller R-value; Berman et al., 2000). We note that because these 3D clustering methods only run on proteins with corresponding structural information, their results may be biased toward known cancer genes (i.e., 55.3% of cancer genes have structural templates whereas 28.1% of all genes have structural templates). Methods are run with default parameters, except GraphPAC and SpacePAC, where the significance threshold (*α*) is set to 1.0 to maximize the number of scored genes returned.

For each method, enrichment for known driver genes on increasingly larger sets of predictions is computed; enrichment is computed at each gene rank on pan-cancer results and at every tenth gene on per-cancer results to reduce the impact of minor reorderings of the relatively small number of known driver genes detected across these datasets. Specifically, we calculate enrichment as the fraction of known driver genes in the gene set (i.e., the precision) divided by the fraction of known driver genes in the whole gene set considered; unmutated driver genes with respect to each mutation dataset are excluded entirely. For this evaluation, we consider the CGC set of known driver genes as a gold standard, as well as alternate positive and negative sets of cancer genes in turn.

Note that LowMACA, NMC, and all three 3D clustering methods did not finish running without error within 30 days on the pan-cancer dataset. We were also unable to run and obtain results from NMC on an additional four individual cancer types (UCEC, SKCM, COADREAD, and LUSC), and thus exclude this method from our evaluation.

## Supplementary Material

2

3

4

5

6

7

## Figures and Tables

**KEY RESOURCES TABLE:** 

REAGENT or RESOURCE	SOURCE	IDENTIFIER
Deposited Data		
InteracDome	Kobren and Singh, 2019	https://interacdome.princeton.edu
Masked somatic exome mutations and RNA-Seq expression data from The Cancer Genome Atlas (TCGA)	Grossman et al., 2016	https://portal.gdc.cancer.gov
Cancer Gene Census	Futreal et al., 2004	https://cancer.sanger.ac.uk/census
UCSC Genome Browser 100-vertebrate alignment	Meyer et al., 2013	http://hgdownload.soe.ucsc.edu/goldenPath/hg38/multiz100way/
Pfam	Finn et al., 2014	https://pfam.xfam.org
1000 Genomes Project	The 1000 Genomes Project Consortium, 2012	https://www.internationalgenome.org/data
Kandoth cancer driver genes	Kandoth et al., 2013, Table S4	https://media.nature.com/original/nature-assets/nature/journal/v502/n7471/extref/nature12634-s1.zip
Lawrence cancer driver genes	Lawrence et al., 2014, Table S2	https://media.nature.com/original/nature-assets/nature/journal/v505/n7484/extref/nature12912-s3.xlsx
Bailey, Tokheim cancer driver genes	Bailey et al., 2018, Table S1	https://ars.els-cdn.com/content/image/1-s2.0-S009286741830237X-mmc1.xlsx
Vogelstein cancer driver genes	Vogelstein et al., 2013, Tables S2A, S2B, S3A–S3C, and S4	http://science.sciencemag.org/highwire/filestream/594203/field_highwire_adjunct_files/1/1235122TablesS1–4.xlsx
Davoli “negative” driver genes	Davoli et al., 2013, Table S2A	https://ars.els-cdn.com/content/image/1-s2.0-S0092867413012877-mmc2.xlsx
DISEASES	Pletscher-Frankild et al., 2015	http://download.jensenlab.org/human_disease_textmining_full.tsv
UniProtKB	The UniProt Consortium, 2019	https://www.uniprot.org/uniprot/
NegAgoFull and NegAgoClean “negative” driver genes	Silverbush et al., 2019; Tables S1D and S1C	https://ars.els-cdn.com/content/image/1-s2.0-S2405471219301474-mmc2.xlsx
Protein Data Bank	Berman et al., 2000	https://www.rcsb.org
Pfam2Go	Mitchell et al., 2015	http://current.geneontology.org/ontology/external2go/pfam2go
Reactome pathways	Jassal et al., 2020	https://reactome.org
Software and Algorithms		
PertInInt	This paper	https://github.com/Singh-Lab/PertInInt
HMMER	Eddy, 2011	http://hmmer.org
DiffMut	Przytycki and Singh, 2017	https://diffmut.princeton.edu\
CanBind	Ghersi and Singh, 2014	http://canbind.princeton.edu
Hotspot	Chang et al., 2016	https://github.com/taylor-lab/hotspots
MutSigCV	Lawrence et al., 2014	https://software.broadinstitute.org/cancer/cga/mutsig_run
OncodriveFML	Melloni et al., 2016	https://bitbucket.org/bbglab/oncodrivefml/src/master/
OncodriveClust	Tamborero et al., 2013	http://bg.upf.edu/group/projects/oncodrive-clust.php
NMC	Ye et al., 2010	https://www.ncbi.nlm.nih.gov/pmc/articles/PMC2822753/bin/1471–2105-11–11-S1.DOC
ActiveDriver	Reimand and Bader, 2013	https://github.com/reimandlab/ActiveDriver
eDriver	Porta-Pardo and Godzik, 2014	https://github.com/eduardporta/e-Driver.git
LowMACA	Melloni et al., 2016	https://bioconductor.org/packages/LowMACA
GraphPAC	Ryslik et al., 2014a	https://bioconductor.org/packages/GraphPAC/
iPAC	Ryslik et al., 2013	https://www.bioconductor.org/packages/iPAC/
SpacePAC	Ryslik et al., 2014a	https://www.bioconductor.org/packages/SpacePAC/
eDriver3D	Porta-Pardo et al., 2015	https://github.com/eduardporta/e-Driver.git
Broad’s GSEA, version 4.0.3	Mootha et al., 2003; Subramanian et al., 2005	https://www.gsea-msigdb.org/gsea/

## Highlights

- Unified framework identifies cancer genes enriched in mutations in functional sites
- Fast analytical calculations obviate the need for time-prohibitive permutation tests
- Integration of structurally resolved interaction site data for >60% of human genes
- Disrupted molecular functionalities revealed in known or newly predicted cancer genes
