# Supplementary material for: PertInInt: An Integrative, Analytical Approach to Rapidly Uncover Cancer Driver Genes with Perturbed Interactions and Functionalities
Source: Cell Syst. Author manuscript; Available in PMC 2020 Sep 16. (PMC7493809; doi:10.1016/j.cels.2020.06.005)
Supplement: 2 [file NIHMS1605104-supplement-2.pdf]

Supplemental Figures

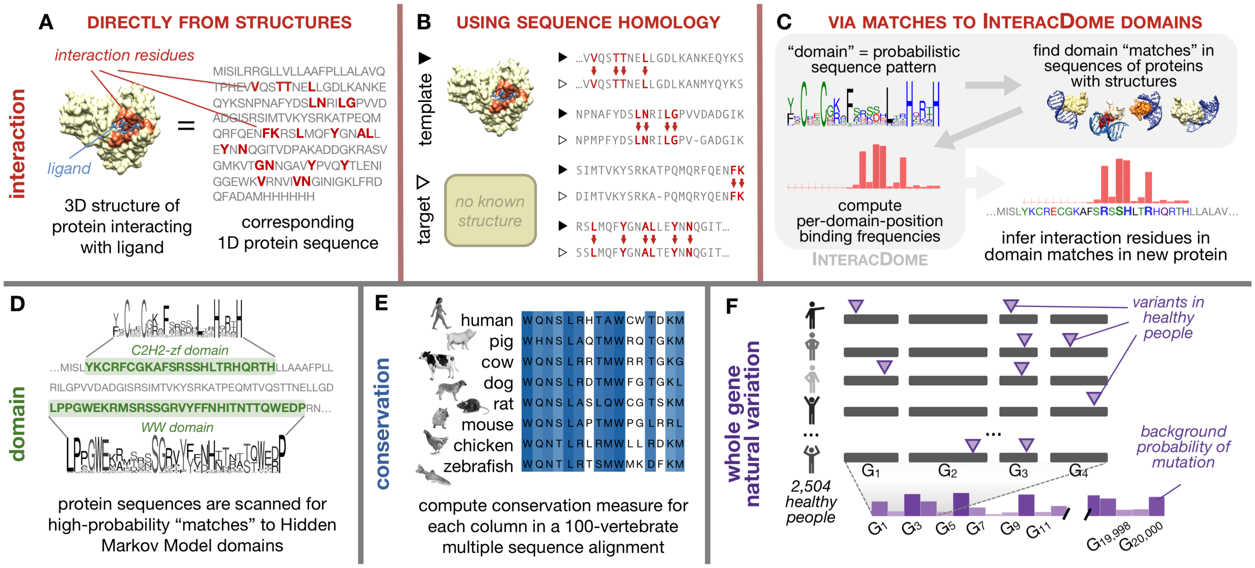

Figure S1: Intuition and data sources behind PertInInt's four track types. ***Related to Figure 1.*** Graphical description of the sources of (A–C) interaction tracks, (D) domain tracks, (E) conservation tracks, and (F) natural variation tracks used by PertInInt. **(A)** Residues within human proteins that contact ligands can be directly determined from a 3-dimensional structure of that protein in complex with a ligand, if such a co-complex structure exists (left). These positions are then marked as "interaction residues" in the corresponding protein sequence (right). **(B)** Interaction residue information from a template protein (depicted by ▶) with a solved co-complex structure can be transferred to a homologous target protein (depicted by ▷) in regions with high sequence similarity, as previously described (Ghersi and Singh, 2014). **(C)** Steps in the shaded box summarize how the previously published InteracDome database (Kobren and Singh, 2019) was generated: matches to protein domains—represented as probabilistic sequence patterns in the form of Hidden Markov Models in Pfam (Finn et al., 2014)—are found in the sequences of proteins that have solved co-complex structures, and then each position within the domain is assigned a "binding frequency" value that corresponds to the fraction of times a residue at that position is found to be in contact with a ligand across co-complex structures. Human protein sequences are scanned for matches to InteracDome domains, and binding frequency information is transferred from the InteracDome domain pattern to the human protein sequence at the site of the domain match (bottom right). **(D)** Human protein sequences are scanned for matches to any Pfam domain. Each domain match generates a new domain track, where protein positions within the domain match region get a score of 1 and protein positions outside get a score of 0. **(E)** For each human protein, a per-position score reflects that position's conservation, computed as previously described (Capra and Singh, 2007) from the corresponding column in a 100-vertebrate multiple sequence alignment. **(F)** Human genes are ranked by the number of variants observed to affect them across a population of healthy individuals and then converted to a background probability of mutation, as previously described (Przytycki and Singh, 2017), to comprise the natural variation tracks.

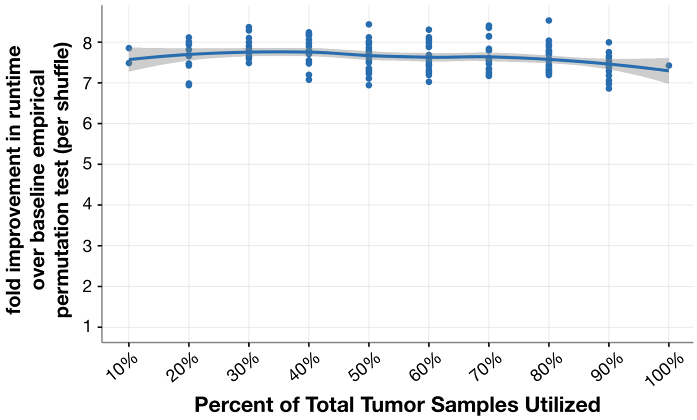

Figure S2: PertInInt's analytical approach results in >7× speedup over baseline empirical permutation approach. ***Related to Figure 1; STAR Methods.*** As a function of the percent (10–100%) of all tumor samples randomly selected from the pan-cancer dataset (*x*-axis), PertInInt's runtime is compared to a baseline version that uses 1,000 empirical permutations of mutations to estimate *Z*-scores for each track. Shown on the *y*-axis is the fold speedup in runtime for ten random selections of tumor samples of each size. The speedup shown is *per permutation* (i.e., divided by 1,000—the total number of permutations performed across each track). The solid blue line represents the local polynomial regression line, with the gray shading showing standard error. Due to the relatively large runtime of the empirical shuffling procedure, these runtime comparisons use only a single track per protein, conservation.

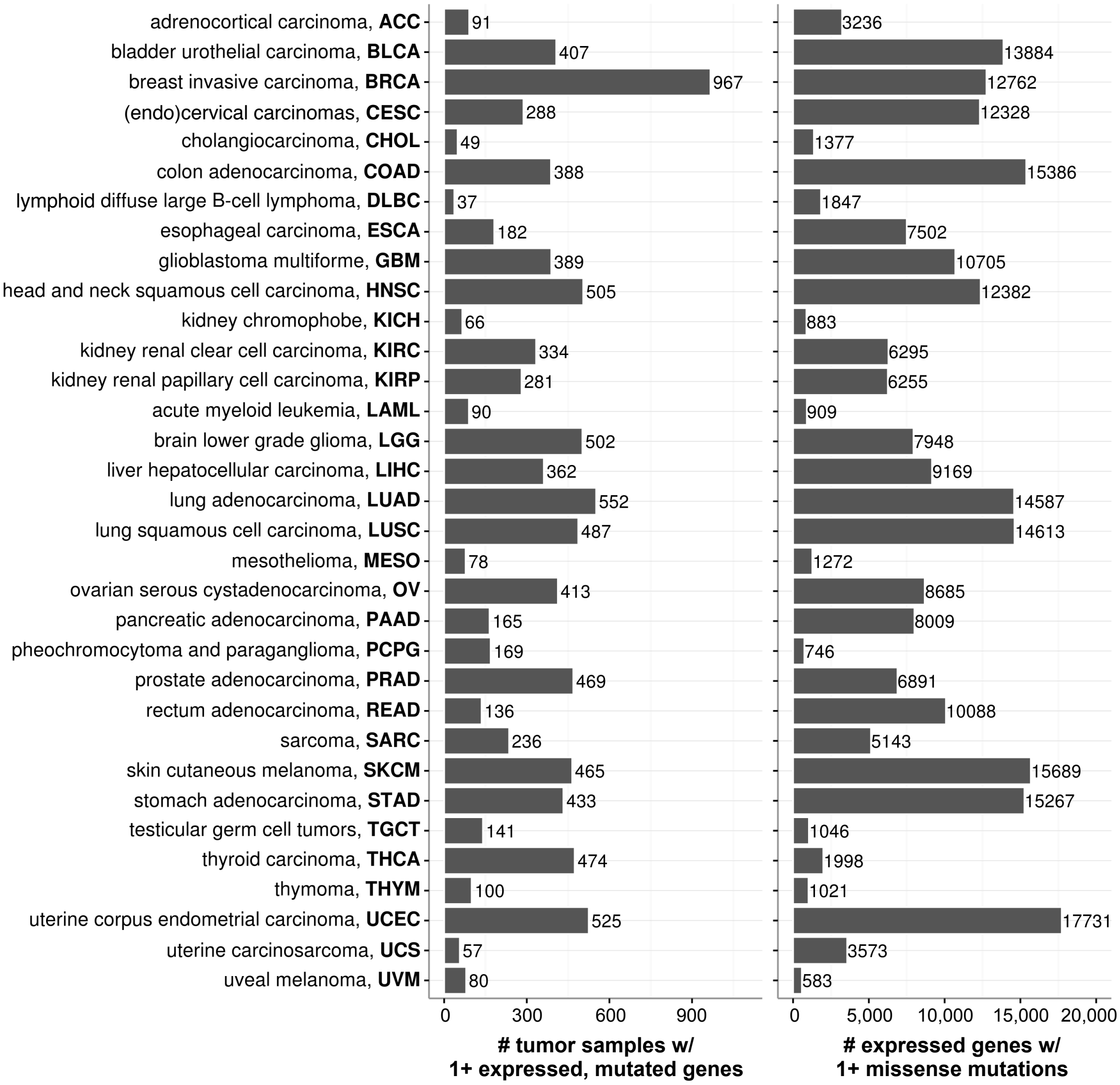

Figure S3: Summary of somatic mutation data. ***Related to Figure 2; Figure 3.*** Somatic mutation data obtained from NCI's Genomic Data Commons Data Portal for 33 cancer types (Fan et al., 2016). The number of tumor samples with 1+ expressed (TPM $\geq$ 0.1) genes with at least one missense mutation is shown in the left plot. The number of genes that are expressed in 1+ tumor samples and have at least one missense mutation is shown in the right plot.

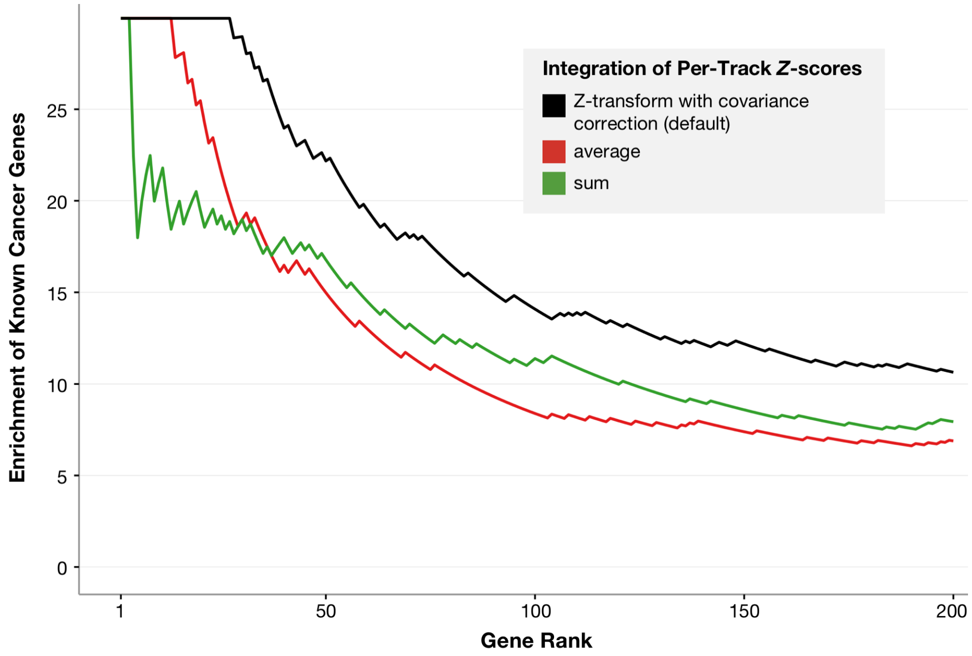

Figure S4: Covariance-based track integration outperforms naïve track integration. ***Related to STAR Methods.*** The default version of PertInInt (black line) combines per-track *Z*-scores using an analytically-computed covariance matrix to account for between-track dependencies. We implemented versions of PertInInt where per-track *Z*-scores are combined using mean (red line) and summation (green line) to generate two new ranked lists of genes on the pan-cancer dataset. Note that these two naïve track integrations are incorrect because they do not account for the dependencies across tracks. For each ranked list of genes, we compute enrichment as the ratio between the fraction of gold standard CGC genes in the top ranked genes (i.e., the precision) and the fraction of CGC genes in the whole set of genes (i.e., the expected precision given a random ordering of genes). All curves converge to an enrichment of 1 by the end of the ranked list of genes (not shown).

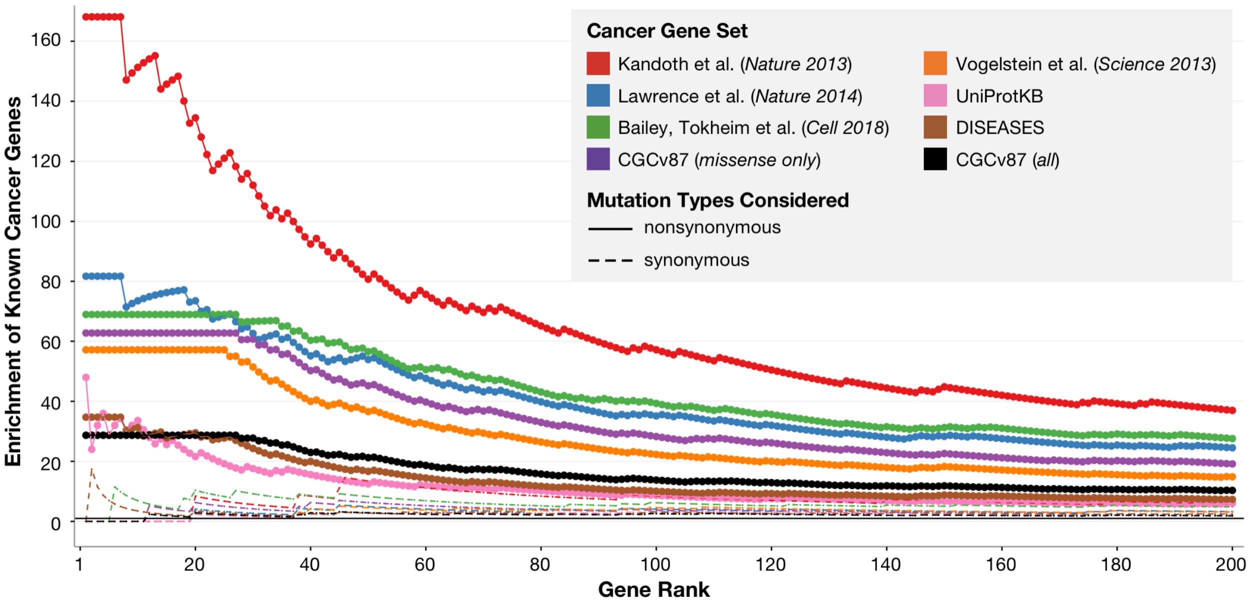

Figure S5: Highly ranked genes are enriched in cancer genes. ***Related to Figure 4; Table S2.*** Gold standard driver gene sets include: 123 genes listed in Kandoth et al., 2013, Table S4 (red), 249 genes listed in Lawrence et al., 2014, Table S2 (blue), 295 genes listed in Bailey et al., 2018, Table S1 (green), all 358 oncogenes and TSGs listed in Vogelstein et al., 2013, Tables S2A-B, S3A-C, S4 (orange), 428 genes from UniProtKB (The UniProt Consortium, 2018) annotated with keywords “oncogene” (KW-0553), “proto-oncogene” (KW-0656) or “tumor suppressor” (KW-0043) (pink), 590 genes from the DISEASES database (Pletscher-Frankild et al., 2015) with confident (i.e., edge weight $>$ 2.75, where the maximum possible edge weight is 5) literature-mined associations with “cancer” (DOID:162) (brown), 713 genes listed in the CGC, version 87 (black), and 324 genes in the CGC with driver statuses due to missense mutations (purple). Ranked gene lists are obtained by applying PertInInt to pan-cancer nonsynonymous mutations (shown as solid lines) and to pan-cancer synonymous mutations (shown as dashed lines). Enrichment for each gold standard set is computed as the ratio between the fraction of gold standard genes in PertInInt’s top ranked genes (i.e., the precision) and the fraction of gold standard genes in the whole set of genes (i.e., the expected precision given a random ordering of genes). All curves converge to an enrichment of 1 by the end of the ranked list of genes (not shown).

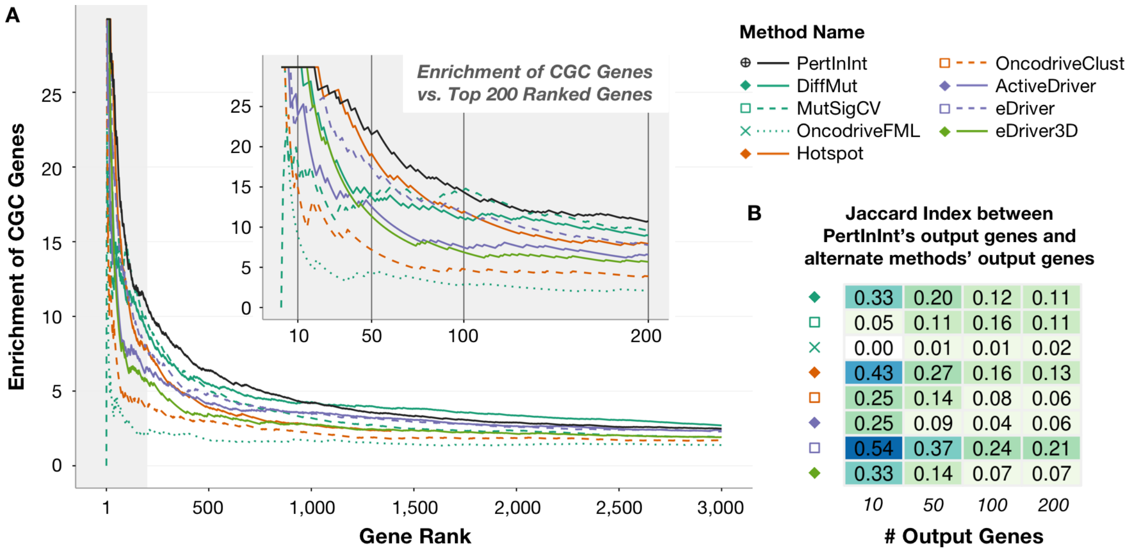

Figure S6: Detection of CGC genes from a pan-cancer dataset excluding highly mutated cancers by PertInInt and alternate methods. ***Related to Figure 4.*** Each driver gene detection method was run on the pan-cancer set of mutations with tumor samples from highly-mutated BLCA, STAD, SKCM, LUAD, LUSC, and ESCA cancers—where there are more than 100 mutations per tumor sample on average—excluded. **(A)** Curves indicate the enrichment for genes in the CGC as we consider an increasing number of output genes for each driver gene detection method. All methods scored at least 3,000 genes except for Hotspot (orange solid line), which only returned 1,397 genes and whose curve ends at that point. The gray shaded area highlights the plot to 200 genes, a closeup of which is shown in the inset. Vertical lines at 10, 50, 100, and 200 ranked genes in the inset correspond to gene set sizes featured in part (B). **(B)** Jaccard Indices (JIs) are calculated between the top 10, 50, 100, and 200 genes output by PertInInt and the corresponding top 10, 50, 100, and 200 genes output by each other method. Lighter colors indicate lower JIs and less overlap between the gene sets.

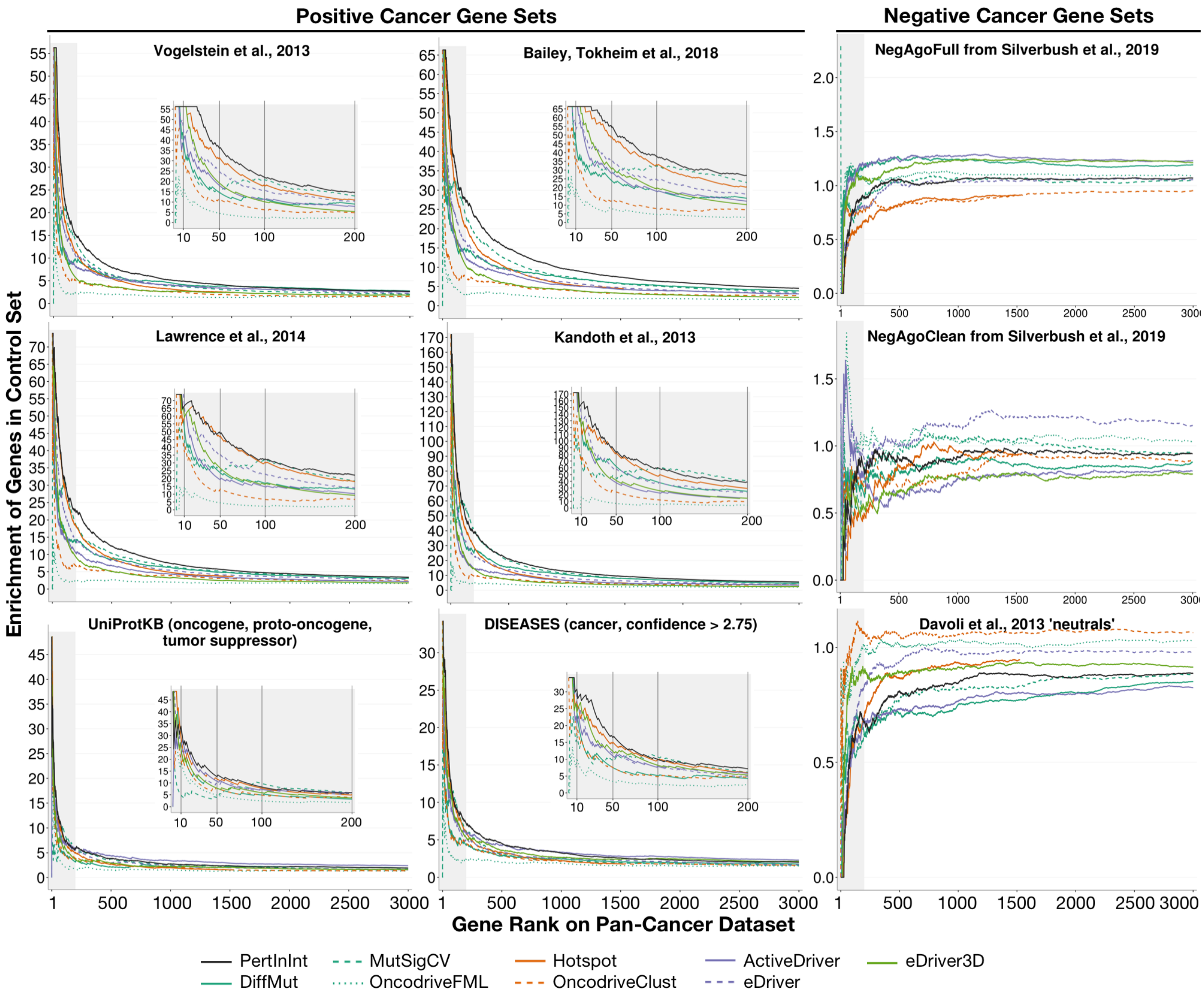

Figure S7: Detection of positive and negative driver genes by PertInInt and alternate methods. ***Related to Figure 4; Table S2; Figure S5.*** Each method was run on the pan-cancer set of mutations as described in STAR Methods. Curves indicate the enrichment for genes in selected positive or negative cancer driver gene sets as we consider an increasing number of output genes for each driver gene detection method. The gray shaded areas highlight each plot to 200 genes, closeups of which are shown in the insets. Positive driver gene sets are described in the caption for Figure S5. Negative driver gene sets include: 8,893 genes that have been proposed to be unlikely to be implicated in cancer and a filtered set of 2,839 of these genes listed in Silverbush et al., 2019, Tables S1D and S1C and 10,303 "neutral" non-driver genes listed in Davoli et al., 2013, Table S2A.

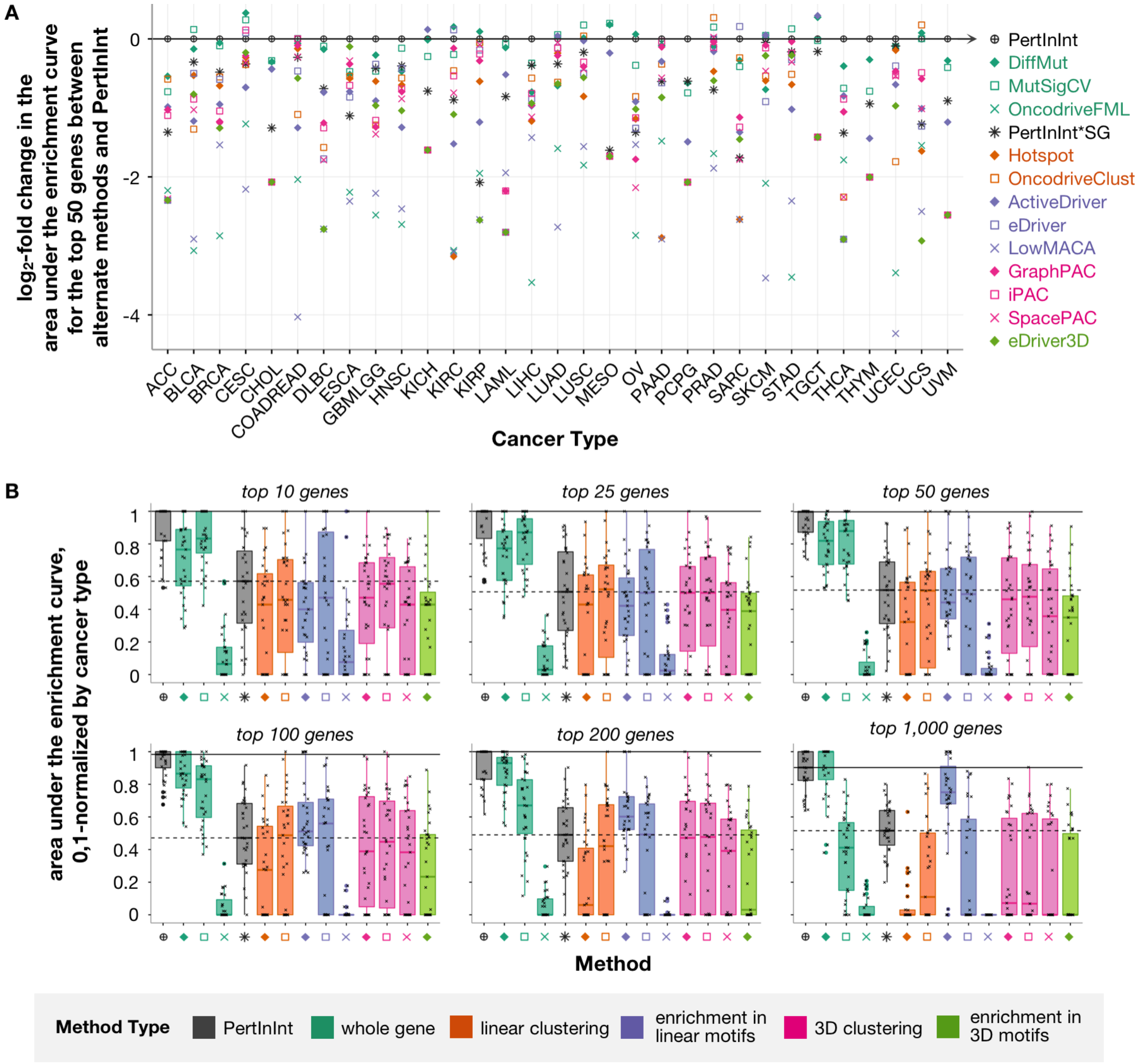

Figure S8: Relative detection of known cancer genes from individual cancer datasets. ***Related to Figure 4.*** **(A)** Log$_2$-fold change between the area under the enrichment curves for the top 50 genes scored by alternate methods and the top 50 genes scored by PertInInt across individual cancer types. "PertInInt*SG" refers to a version of PertInInt where only subgene resolution tracks are included. PertInInt tends to perform better than the alternate methods, as most of these values are below 0. **(B)** For each cancer type, the areas under the enrichment curves computed for the top 10 (or 25, 50, 100, 200, or 1,000) genes ranked by each driver gene detection method are linearly scaled to fall between 0 and 1. For example, when looking at the top 50 genes ranked by each method when run on SARC mutations, Hotspot has the relatively smallest area under the enrichment curve and thus gets a scaled value of 0, whereas PertInInt has the relatively largest area under the enrichment curve and thus gets a scaled value of 1. Then for each computational method, a box plot of their corresponding values across cancer types is shown. Jittered data points representing different cancer types are overlaid on boxplots. Horizontal solid and dashed lines are drawn at the median relative area under the enrichment curve for PertInInt and PertInInt*SG respectively in each plot. Methods are labeled as in (A).

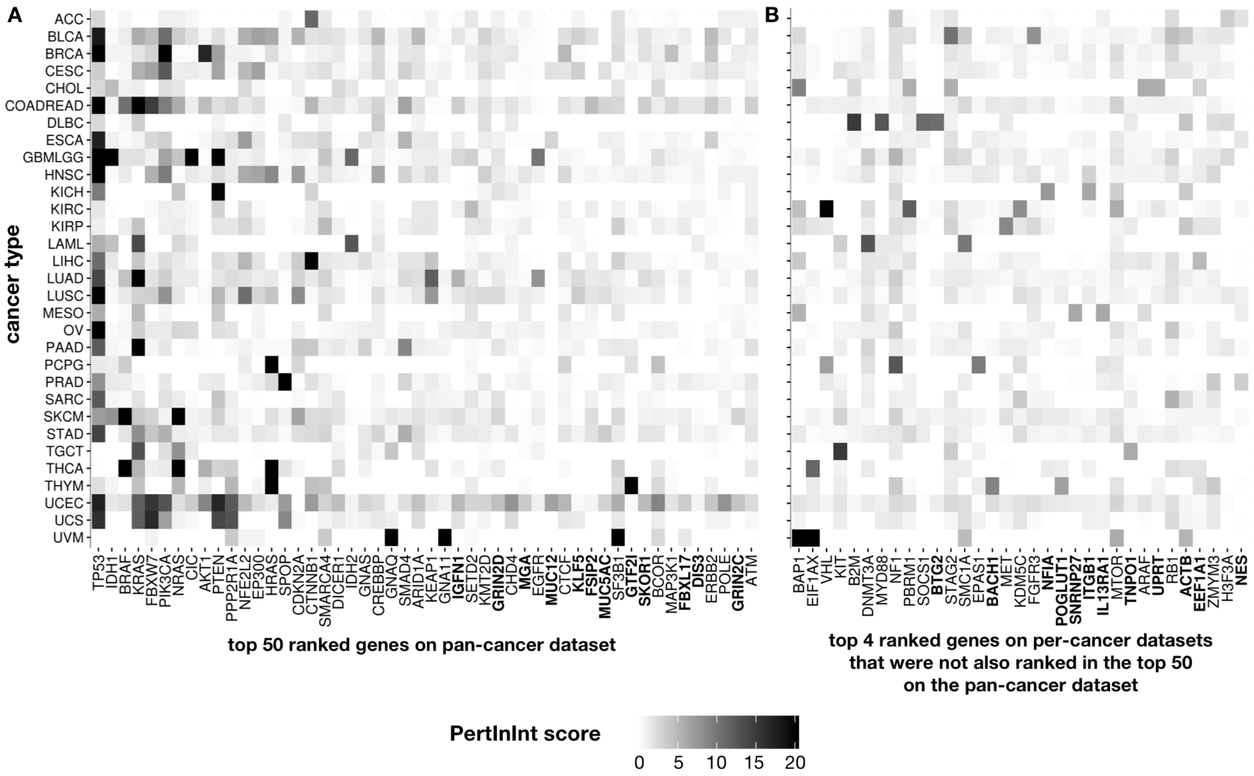

Figure S9: Distinct cancer-relevant genes are highly ranked in individual cancer datasets. *Related to Figure S8.* Each entry corresponds to a gene–cancer pair and is colored by the PertInInt score of that gene (genes listed along the x-axis) when run on data from the corresponding cancer type individually (cancer types listed along the y-axis). All PertInInt scores $\geq$20 are recorded as 20 for visualization purposes. Genes that are *not* in the CGC are bolded in the *x*-axis. **(A)** Top 50 genes ranked by PertInInt when run on the pan-cancer dataset. **(B)** Genes that are ranked within the top four by PertInInt when run on individual per-cancer datasets, but are not found in the top 50 genes when PertInInt is run on the pan-cancer dataset.

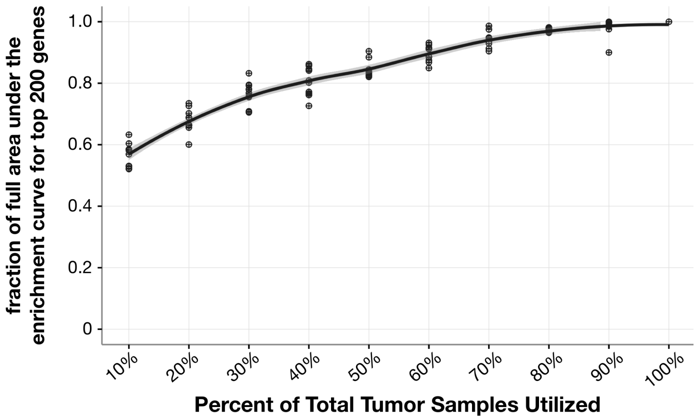

Figure S10: PertInInt's power increases with more tumor samples. ***Related to STAR Methods.*** As a function of the percent (10–100%) of all tumor samples randomly selected from the pan-cancer dataset (x-axis), we show the area under the enrichment curve for the top 200 genes scored by PertInInt when run on each tumor sample subset, normalized by the area under the enrichment curve for PertInInt's top 200 predictions when using all tumor samples (y-axis). Ten random selections of samples are analyzed at each sample size. The solid black line represents the local polynomial regression line of these normalized areas under the enrichment curve with respect to the sample size. PertInInt's ability to recapitulate cancer genes increases with sample size.

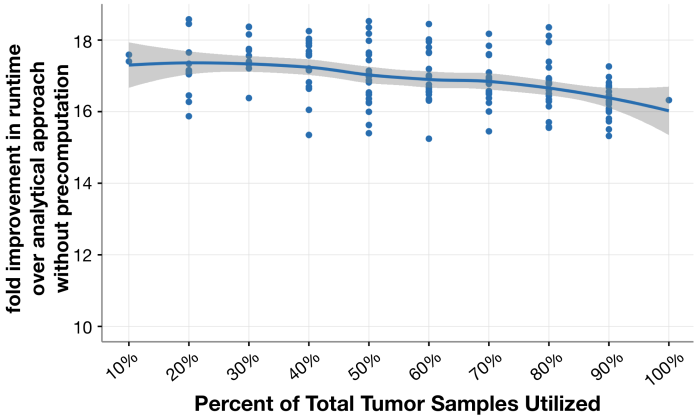

Figure S11: Precomputation enables $>16\times$ speedup over basic analytical approach. ***Related to Figure 1; Figure S2; STAR Methods.*** As a function of the percent (10–100%) of all tumor samples randomly selected from the pan-cancer dataset (x-axis), PertInInt's runtime is compared to a baseline version that does not use precomputed expectation and variance estimates to compute Z-scores for each track. Shown on the y-axis is the fold speedup in runtime for ten random selections of samples of each size. The solid blue line represents the local polynomial regression line, with the grey shading showing standard error. These runtime comparisons use only a single track per protein, conservation, as in Figure S2.
